# SNPEVG: a graphical tool for GWAS graphing with mouse clicks

**DOI:** 10.1186/1471-2105-13-319

**Published:** 2012-11-30

**Authors:** Shengwen Wang, Daniel Dvorkin, Yang Da

**Affiliations:** 1 Department of Animal Science, University of Minnesota, St. Paul, USA

## Abstract

**Background:**

Genome-wide association studies (GWAS) using single nucleotide polymorphism (SNP) markers generate large quantities of tests results. Global and local graphical viewing of the test results is an effective approach to digest and interpret GWAS results.

**Results:**

SNPEVG is a set of graphical tools for instant global and local viewing and graphing of GWAS results for all chromosomes and for each trait. The current version includes three programs, SNPEVG1, SNPEVG2 and SNPEVG3. SNPEVG1 is a graphical tool for SNP effect viewing of P-values allowing multiple traits. The total number of graphs that can be generated by one ‘Run’ is n(c + 2), where n is number of ‘traits’ with 0 < n ≤ 100, and c is the number of chromosomes. SNP effect viewing and graphing is accomplished through a user friendly graphical user interface (GUI) that provides a wide-range of options for the user to choose. The GUI can produce the Manhattan plot, the Q-Q plot of all SNP effects, and graphs for SNP effects by chromosome by clicking one command. Any or all the graphs can be saved with publication quality by clicking one command. SNPEVG2 is for the viewing and graphing of multiple traits on the same graph with options to graph any or all of the traits, customizable colors and user specified Y1 or Y2 axis for each traits. The SNPEVG3 program uses the output file of single-locus test results from the epiSNP computer package as the input file. Each chromosome figure can display three genetic effects (genotypic, additive and dominance effects), and the number of observations.

**Conclusions:**

The SNPEVG package is a versatile, flexible and efficient graphical tool for rapid digestion of large quantities of GWAS results with mouse clicks.

## Background

GWAS analysis generally yields large quantities of test results. Global and local graphical viewing of the test results is an effective approach and often is a necessary step for interpreting GWAS results. A widely used graphical viewing of GWAS results is the Manhattan plot, which provides a global graphic view of GWAS results of all chromosomes for a trait on one graph to quickly identify genome locations with the most significant SNP effects [1,2]. Following this global view, detailed graphical examination of each chromosome is helpful for further understanding the GWAS results, and more graphical work often is needed for effective presentation of the GWAS results. The purpose of the SNPEVG package is to provide a graphical tool for rapid digestion of GWAS results and to accomplish large quantities of graphical tasks of GWAS analysis in a seamless fashion.

## Implementation

The SNPEVG computer package is implemented in the C++ programming language. The object orientation feature of the C++ language enables the efficient software development cycle by easy reuse of modules for different applications with similar features. The SNPEVG computer package used the Qt library under the terms of the GNU Lesser General Public License (LGPL) version 2.1 as shown [3].

## Results and discussion

SNPEVG Version 3.2 includes three graphical programs: SNPEVG1, SNPEVG2 and SNPEVG3. SNPEVG1 is for graphing effects of one trait per graph for up to 100 traits, SNPEVG2 is for graphing multiple traits on the same graph, and SNPEVG3 processes directly uses an output file of EPISNP or EPISNPmpi [2] as the input file. Both SNPEVG1 and SNPEVG2 using the same format of the input file, which contain name, chromosome number and chromosome position of each SNP marker, and P-values of statistical tests from any method. Each program has a scalable GUI allowing efficient and flexible use of computer screen and allows the production of graphical images with user defined vertical/horizontal ratios. Each program can be launched multiple times by mouse click of the executable program so that the user can compare graphical effects of different graph options simultaneously. SNPEVG 3.2 is available from Additional files 1 and 2 or from the website at http://animalgene.umn.edu. Full features of the SNPEVG package are described in the SNPEVG user manual [4] Additional file 3.

### The SNPEVG1 program

**Figure 1:**
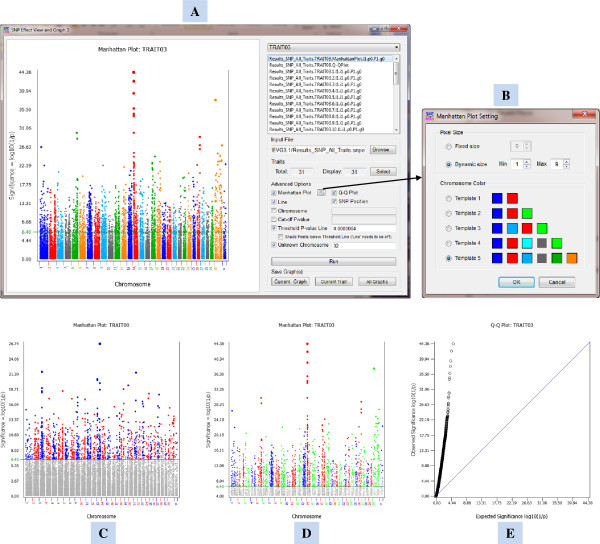
**SNPEVG1 GUI and Manhattan and Q-Q plots for global viewing and graphing of GWAS results. A**: The user friendly GUI of SNPEVG1 offers user interactive viewing and graphing of global and local test results. **B**: The ‘Manhattan setting’ plate for customized chromosome color, fixed pixel size, or dynamic pixel size proportional to P-values in Manhattan plot. **C**: A Manhattan plot with color Template 1, proportional pixel size, and shading of P-values below the threshold P-value line. **D**: A Manhattan plot with color Template 2, proportional pixel size, and elimination of P-values below the line of cut-off P-value. **E**: A Q-Q plot.

**Figure 2:**
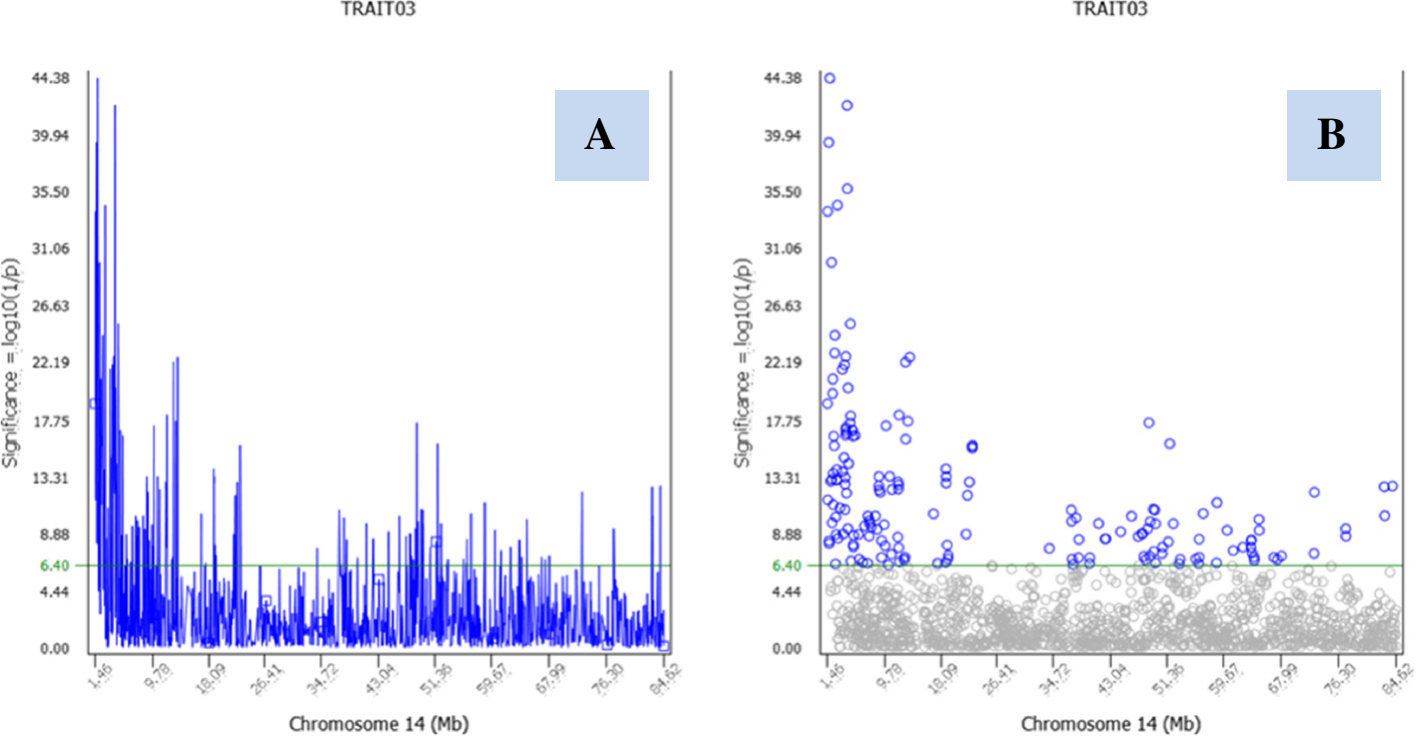
**Chromosome graphs of SNPEVG1. A**: Chromosome figure of SNPEVG1 with connecting line between adjacent data points. **B**: Chromosome figure of SNPEVG1 without connecting line between adjacent data points.

SNPEVG1 supports a maximum 100 traits. The GUI (Figure 1A) has numerous graphical options for Manhattan plots, including user-customized colors (Figure 1B), shading P-values below the threshold P-value line (Figure 1C), and scalable pixel size proportional to P-values [5] (Figure 1A 1C), and displaying P-values above the specified cut-off P-value (Figure 1D). Each Manhattan plot uses true chromosome size defined by the starting and ending SNP marker positions of the chromosome. P-values for the unknown chromosome are displayed in sequential order of SNP markers rather than chromosome positions. Manhattan plots and Q-Q plots (Figure 1E) provide global view of test results for each trait. In addition to global viewing, the GUI produces graphs for each chromosome and each trait. For each chromosome, P-values can be presented as connected lines (Figure 2A) or separate symbols (Figure 2B). The total number of graphs that can be generated is n(c + 2), where n is the number of ‘traits’ with 0 < n ≤ 100, and c is the number of chromosomes. Assuming 30 traits and 30 chromosomes per trait, the program produces 960 graphs for interactive viewing by one click of ‘run’, including 30 Manhattan plots, 30 Q-Q plots and 900 chromosome graphs. The upper-right window of the GUI (Figure 1A) is the ‘Graph list’ by trait, showing a list of graphs produced by the ‘Run’ button. The user can turn off Manhattan and Q-Q plots, scroll the chromosome graphs of each trait using the up or down arrow key, and switch between traits using the left or right key. Any selected graph, or graphs for selected traits, or all graphs can be saved as graphical images with publication quality by clicking a button on the GUI. SNPEVG1 requires a simple text input file with the following columns: CHR, POSITION, SNP, and P-VALUE columns, where CHR = chromosome number, POSITION = chromosomal position of the SNP marker, SNP = name of the SNP marker, and P-VALUE is the P-value for a trait.

### The SNPEVG2 program

**Figure 3:**
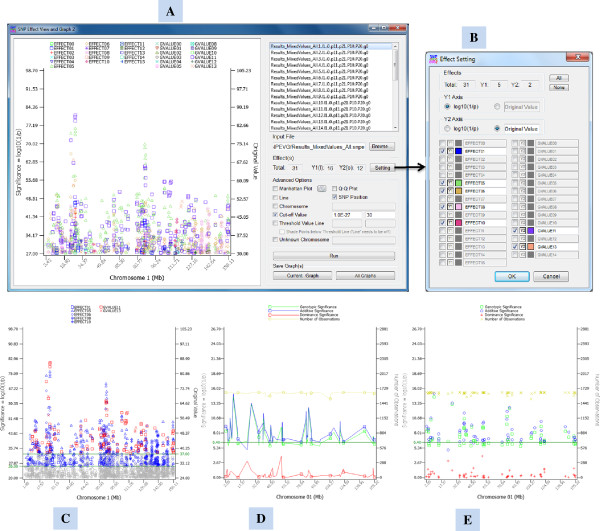
**SNPEVG2 program GUI and chromosome graphs of SNPEVG2 and SNPEVG3. A**: The user friendly GUI of SNPEVG2 offers user interactive viewing and graphing of global and local test results from multiple traits on the same graphs. **B**: The ‘Effect setting’ plate for the selection of traits to be displayed, the selection of Y1 or Y2 axis, and customized chromosome color for each trait. **C**: A chromosome graph with two threshold value lines for Y1 and Y2 axes, and elimination of P-values below the line of cut-off P-value line. **D**: Chromosome figure of SNPEVG3 with connecting line between adjacent data points. **E**: Chromosome figure of SNPEVG3 without connecting line between adjacent data points.

SNPEVG2 is designed to display P-values of multiple traits on the same graph. Each chromosome figure can display P-values in log scale or the original values of a variable on either Y1 or Y2 axis (up to 100 traits) (Figure 3A). The Y2 axis can be used to display a variable unrelated to P-values such as minor allele frequency or allele frequency difference between the best and worst individuals, allowing the production of more flexible and informative graphs than using Y1 axis presenting P-values only. The chromosome graphs can be crowded and difficult to view if the number of traits is large. This problem can be solved by the option to select traits to display, to customize the color of each trait or switch Y1 and Y2 axes using the ‘Setting’ button on the GUI (Figure 3B). Each Y axis, Y1 or Y2, can have its own threshold P-value or cut-off P-value (Figures 3A and C). SNPEVG2 requires a simple text input file with the same format as for SNPEVG1, i.e., CHR, POSITION, SNP, and P-VALUE columns, where CHR = chromosome number, POSITION = chromosomal position of the SNP marker, SNP = name of the SNP marker, and P-VALUE is the P-value for a trait.

### The SNPEVGconvert program

The SNPEVGconvert program is designed to convert an output file from any GWAS analysis software to the format of SNPEVG1 and SNPEVG2. With this format conversion program, virtually any GWAS software could SNPEVG1 and SNPEVG2. To use this program, the user only needs to specify the number of columns in the original files and identify the column numbers to be printed in the input file for SNPEVG1 and SNPEVG2.

### The SNPEVG3 program

SNPEVG3 is developed for graphical analysis of GWAS using the output file of single-locus test results of EPISNP or EPISNPmpi [2] as the input file for drawing figures. SNPEVG3 has similar GUI features as SNPEVG1, but it does not have the limit of 100 traits. This program draws graphs for P-values of additive, dominance and genotypic effects on the Y1 axis and draws sample size on the Y2 axis. The P-values can be displayed with lines connecting adjacent data points (Figure 3D) or use symbols without connecting lines (Figure 3E). The user has an option to draw a figure by a sorted effect such as additive or dominance effect.

### Evaluation of sample size limitations

Currently, SNPEVG1, SNPEVG2 and SNPEVG3 have a Microsoft Windows 32-bit version and a 64-bit version for Mac OS X 10.6 or newer. A Windows 64-bit version is expected to become available at a later time. For practical purposes, either the 32-bit or the 64-bit version would be powerful enough for real GWAS data sets. For a single trait, the 32-bit version could process 10 million markers per trait in about 30 seconds but failed for 12 million markers, and the 64-bit version could process 30 million markers in 80.62 seconds (Table 1). For multiple traits, the number of markers that can be processed per trait is approximately the numbers in Table 1 divided by the number of traits (Table 2).

**Table 1:** Processing time and limit of SNP markers for SNPEVG1 and SNPEVG3 of 32-bit (Windows 7) and 64-bit versions (Mac OS X) for one trait

**Number of SNPs**	**SNPEVG1 32-bit**	**SNPEVG1 64-bit**	**SNPEVG3 32-bit**	**SNPEVG3 64-bit**
**(N)**	**Time (sec)**	**Time (sec)**	**Time (sec)**	**Time (sec)**
1,000,000	3.08	2.82	4.17	3.32
2,000,000	5.88	4.75	7.67	5.92
3,000,000	8.54	6.78	10.90	8.57
5,000,000	13.67	10.52	17.71	13.11
7,000,000	19.17	14.41	24.59	17.72
10,000,000	26.71	20.11	34.91	25.76
13,000,000	Fail	25.92	Fail	33.49
17,000,000	Fail	33.85	Fail	43.12
21,000,000	Fail	43.50	Fail	54.24
25,000,000	Fail	51.50	Fail	67.84
30,000,000	Fail	61.70	Fail	80.62

The 32-bit version was run on a desktop PC with Microsoft Windows 7 and Intel Core i7-2600 CPU of 3.40 GHz and 8 GB memory. The 64-bit version was run on the MacBook Pro with Mac OS X 10.7 and Intel Core i7 CPU of 2.2 GHz and 8 GB memory.

**Table 2:** Processing time and limit of SNP markers for SNPEVG1 and SNPEVG2 of 32-bit (Windows 7) and 64-bit versions (Mac OS X) for 100 traits

**Number of SNPs**	**SNPEVG1 32-bit**	**SNPEVG1 64-bit**	**SNPEVG2 32-bit**	**SNPEVG2 64-bit**
**(N)**	**Time (sec)**	**Time (sec)**	**Time (sec)**	**Time (sec)**
10,000	1.56	1.57	1.75	1.41
20,000	2.57	2.21	2.36	1.78
30,000	3.75	2.73	2.96	2.17
50,000	5.74	4.17	4.61	2.75
70,000	8.38	5.78	5.80	3.49
100,000	11.79	8.29	8.09	4.75
130,000	Fail	9.23	9.99	6.18
170,000	Fail	13.24	13.26	7.82
210,000	Fail	15.90	Fail	9.09
250,000	Fail	18.93	Fail	11.14
300,000	Fail	23.10	Fail	13.11
350,000	Fail	27.35	Fail	14.96
400,000	Fail	31.86	Fail	17.07

The 32-bit version was run on a desktop PC with Microsoft Windows 7 and Intel Core i7-2600 CPU of 3.40 GHz and 8 GB memory. The 64-bit version was run on the MacBook Pro with Mac OS X 10.7 and Intel Core i7 CPU of 2.2 GHz and 8 GB memory.

## Conclusions

The SNPEVG package is a versatile and efficient graphical tool for rapid digestion of large quantities of test results from GWAS and can be customized for graphical viewing and drawing of non-GWAS information such as allele frequency differences.

## Availability and requirements

Project name: SNPEVG

Project homepage: http://animalgene.umn.edu/

Operating system(s): Microsoft Windows 7, Mac OS X 10.6 or newer

Other requirements: none.

License: none.

Any restrictions to use by non-academics: none.

## Abbreviations

GWAS: Genome-wide association study; SNP: Single nucleotide polymorphism.

## Competing interests

The authors declare that they have no competing interests.

## Authors’ contributions

SW is the author of SNPEVG1, SNPEVG2, and SNPEVG3. DD is the author of the EPISNPPLOT program that is partially used in SNPEVG3. YD designed most functions of the computing tools, and is the lead writer of the manuscript. All authors read and approved this manuscript.

## Supplementary Material

Additional file 1 SNPEVG_Win_3.2.zip: contains all executables and libraries for Microsoft Windows platforms and the user manual of SNPEVG package version 3.2. Click here for file

Additional file 2 SNPEVG_Mac_3.2.zip: contains all executables for Mac OS X platforms and the user manual of SNPEVG package version 3.2. Click here for file

Additional file 3 **A Graphical Tool For SNP Effect Viewing and Graphing.** (PDF 6838 kb) Click here for file
