# Supplementary material for: SNPEVG: a graphical tool for GWAS graphing with mouse clicks
Source: BMC Bioinformatics. 2012 Nov 30;13:319. doi: 10.1186/1471-2105-13-319 (PMC3527193; doi:10.1186/1471-2105-13-319)
Supplement: Additional file 1 — SNPEVG_Win_3.2.zip: contains all executables and libraries for Microsoft Windows platforms and the user manual of SNPEVG package version 3.2. [file 1471-2105-13-319-S1.zip › SNPEVG_user_manual_3.2.pdf]

SNPEVG

# A Graphical Tool For SNP Effect Viewing and Graphing

**Part I: SNPEVG1, page 3**
**Part II: SNPEVG2, page 15**
**Part III: SNPEVGconvert, page 32**
**Part IV: SNPEVG3, page 35**

USER MANUAL VERSION 3.2

---

Shengwen Wang, Daniel Dvorkin, Yang Da

Department of Animal Science
University of Minnesota

November 2012

# Part I: SNPEVG1

## Multiple traits, one trait/graph

## Any method of significance testing

## Contents


1 INTRODUCTION ..........3

2 INSTALLATION ..........4

3 INPUT FILE FORMATS ..........4

4 RUNNING SNPEVG1 ..........5

# 1 INTRODUCTION

SNPEVG1 is a graphical tool for SNP effect viewing and graphing for rapid digestion of large quantities of genome-wide significance test results using P-values from any methods of significance testing. Assuming 30 traits and 30 chromosomes per trait, one command of 'Run' produces 960 graphs that will greatly facilitate the digestion of GWAS results. The total number of graphs that can be generated by one 'Run' is n(c + 2), where n is number of 'traits' with $0 < n \leq 100$, and c is the number of chromosomes. Any of the graphs could be readily placed in a publication.

SNP effect viewing and graphing is accomplished through a user friendly graphical user interface (GUI) that provides a wide-range of options for the user to choose. The GUI can produce the Manhattan plot [1], the Q-Q plot [2] of all SNP effects, and graphs for SNP effects by chromosome by clicking one command. Any or all the graphs can be saved as high resolution png files by clicking one command. The user can also increase the png resolution by using full screen. For example, the default screen size give a 667x667 png resolution and full screen gives 1119x1119 for a 1900x1200 screen resolution. The program allows users to select traits for graphing and viewing. The user also has the options to draw graphs using lines or symbols, or specify a cut-off p-value to be shown on each graph or threshold p-value line for SNP effects to be included in the graphs. The main features and options of SNPEVG1 can be summarized as follows.

- Instant graphics of large quantities of genome-wide significance testing results from any methods for any or all traits.
- Input format conversion from output file of any GWAS software to the input file format for SNPEVG.
- Immediate viewing of all graphics within each SNPEVG1 GUI.
- Scalable SNPEVG1 GUI allowing efficient and flexible use of computer screen.
- Graphics of Manhattan plot, Q-Q plot, and P-value figures for all chromosomes and all traits (up to 100 traits) by one click of 'run'.
- Save of one graph, all graphs for all chromosomes of a trait, or all graphs for all chromosomes and all traits (up to 100 traits) by one of 'Current Graph', 'Current Trait' or 'All Graphs'.
- Each Manhattan plot has the following options and features:
    - Each chromosome width is the true width of the chromosome defined by the starting and ending SNP marker positions;
    - Adjustable fixed pixel size and dynamic pixel size;
    - Color templates with the support of custom colors;
    - Horizontal line marking the threshold P-value for declaring significance;
    - Display of P-values above the specified cut-off P-value;
    - Shading P-values below the threshold P-value line.
- Each chromosomal figure of P-values has the following options:
    - Horizontal line marking the threshold P-value for declaring significance;
    - Display of P-values above the specified cut-off P-value;
    - Shading P-values below the threshold P-value;
    - Optional use of lines to connect data points;
    - User specified unknown chromosome that will plot P-values in the sequential order of SNP markers rather than chromosome positions.
- Q-Q plot for each trait displaying all data points.

# 2 INSTALLATION

## 2.1 Windows x86 / x64

The installation of SNPEVG1 only requires to create a folder, to place all files in SNPEVG.zip in that folder, and then change SNPEVG1_exe file to SNPEVG1.exe file. The Windows x64 64-bit version is available by request.

## 2.2 Mac OS X 10.6 or newer 64-bit

The installation of SNPEVG1 only requires to unzip SNPEVG1_Mac.zip file to obtain SNPEVG1.app file.

# 3 INPUT FILE FORMATS

One input file with the extension 'snpe' for p-values is required. The format of the input file is:

Column 1: SNP name
Column 2: chromosome number
Column 3: SNP position on the chromosome
Columns 4 through Column (n+3): 'Trait columns' of P-values for trait 1 through trait n, where $0 < n \leq 100$.

As an example, the following is an input file named 'SNPEVG1_input1.snpe':

```
SNPID   Chr  Position  T1-geno   T1-add    T1-dom    T2-geno   T2-add    T2-dom
SNP01    1   571340    2.48E-03  1.11E-03  1.58E-03  2.74E-02  2.65E-01  1.11E-03
SNP02    1   845494    7.02E-04  9.07E-04  8.91E-05  2.56E-02  8.02E-02  9.07E-04
SNP03    1   883895    1.87E-02  1.71E-01  3.07E-03  1.07E-02  2.81E-03  1.71E-01
SNP04    1   929617    1.79E-03  1.57E-02  1.39E-05  9.03E-02  2.99E-04  1.57E-02
SNP05    1   950841    1.20E-03  7.07E-03  1.32E-05  8.46E-02  1.71E-03  7.07E-03
```

In the above file, column 1 is the marker name, column 2 is the chromosome number, column 3 is the marker position in units of base pairs (bp) or genetic distance in centi-Morgans, and column 4 through column 9 are P-values of genotypic effect (geno), additive effect (add) and dominance effect (dom) for each trait (T1 or T2 in this example). Any type of user defined genetic effect could be included as a 'trait' column. For example, one type of genetic effect such as additive effects for all traits could be placed in one input file, as shown in the following example named 'SNPEVG1_input2.snpe':

```
SNPID   Chr  Position  T1-add    T2-add    T3-add    T4-add    T5-add
SNP01    1   571340    2.48E-03  1.11E-03  1.58E-03  2.74E-02  2.65E-01
SNP02    1   845494    7.02E-04  9.07E-04  8.91E-05  2.56E-02  8.02E-02
SNP03    1   883895    1.87E-02  1.71E-01  3.07E-03  1.07E-02  2.81E-03
SNP04    1   929617    1.79E-03  1.57E-02  1.39E-05  9.03E-02  2.99E-04
SNP05    1   950841    1.20E-03  7.07E-03  1.32E-05  8.46E-02  1.71E-03
```

For the given input file, SNPEVG1 plots the log10(1/p) values according to the SNP positions on each chromosome for SNP markers with known chromosome positions, or in sequential orders of the SNP markers for SNPs in the chromosome file specified as 'unknown' by the user. For each 'trait' column, SNPEVG1 produces the following graphs:

- Manhattan plot with options;
- Q-Q plot with one option;
- Graphs for one or all chromosomes with options.

## 4 RUNNING SNPEVG1

To run SNPEVG1, open the folder that holds the SNPEVG1 files and required input files. Double click 'SNPEVG1.exe' (For Mac users, double click 'SNPEVG1.app') will open the GUI of SNPEVG1 as shown in Figure 1 below (Figures in the manual are produced in Windows 7):

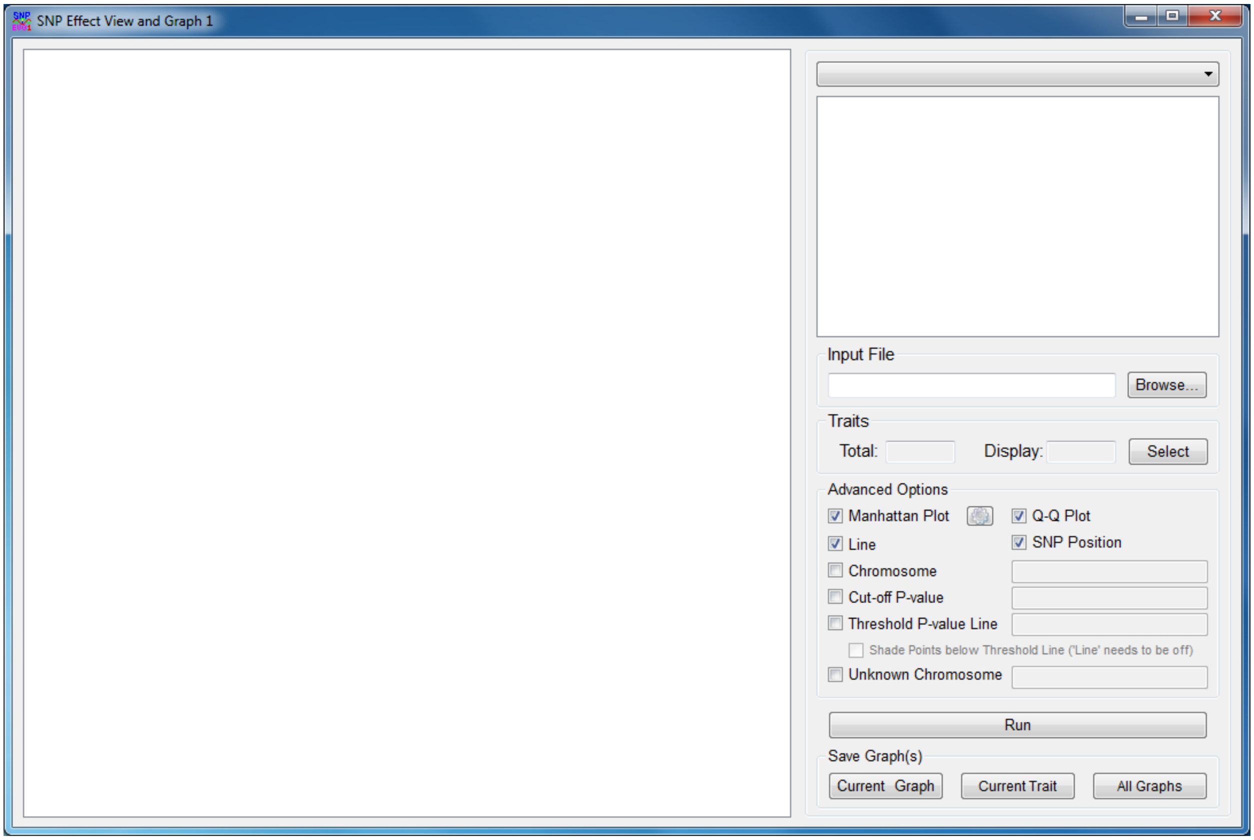


**Figure 1. Graphical User Interface (GUI) of SNPEVG1.**

The GUI shown above is scalable, i.e., the GUI size can be changed by dragging the borders of the screen, so that the GUI can use the full screen or part of the screen, allowing efficient and flexible use of the computer screen.

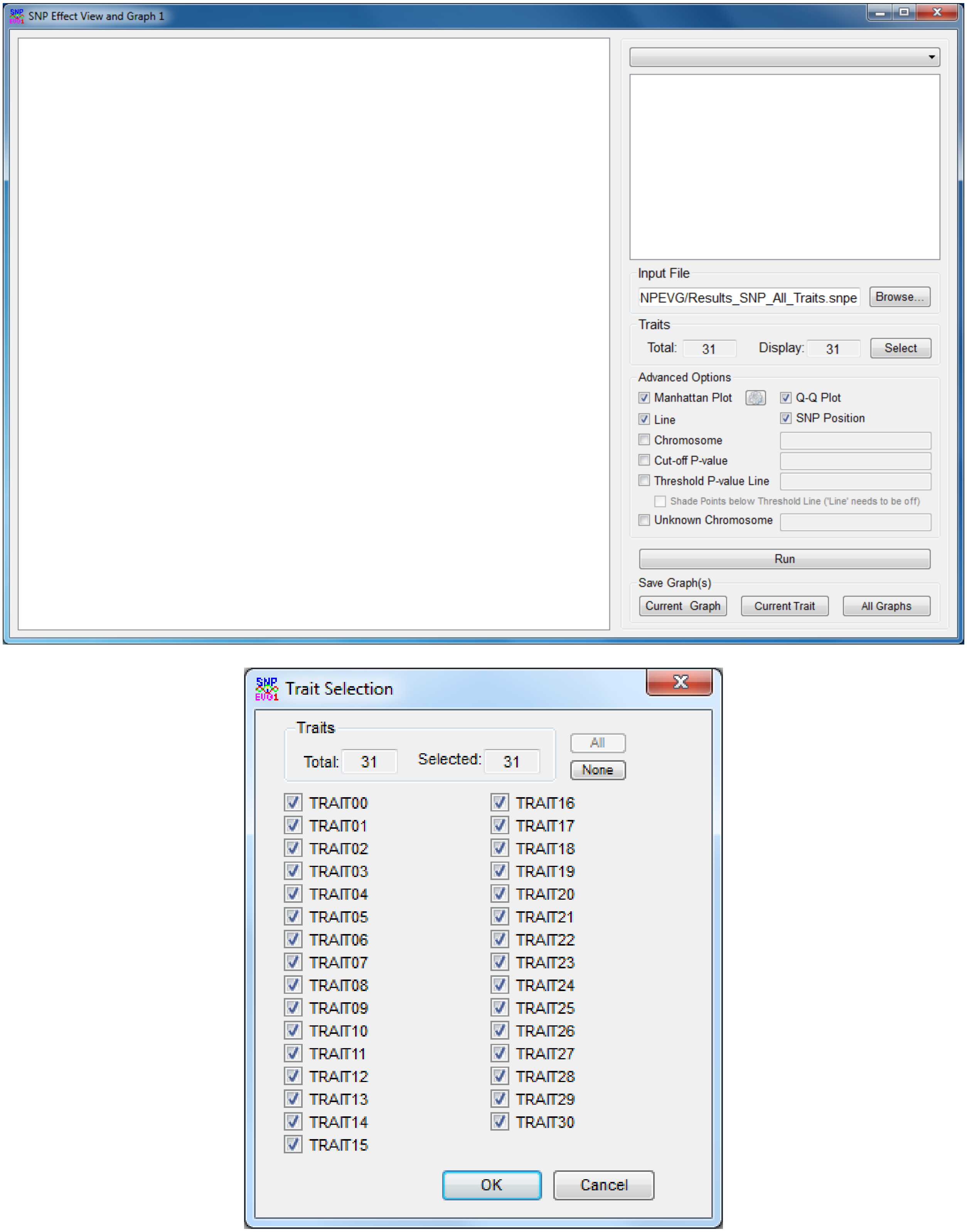


**Figure 2. Upload of an input file (upper) and selection of traits to display (lower).**

### 4.1 Upload Input File, Select Options, and 'Run'

In the GUI of **Figure 1**, upload an input file by clicking 'Browse…', select the options, and then click 'Run' to produce the corresponding graphs. Alternatively, the user can drag the SNPE input file and drop to the input field without using browse button. This program supports maximum 100 traits for graphics display, and the extra traits in the input file are discarded. When the input file is specified, total number of traits and number of selected traits are displayed as shown in the upper figure in **Figure 2**. All traits are selected by default. Clicking 'Select', a dialog in the lower figure in **Figure 2** pops up and allows the user to select the traits that will be displayed.

### 4.2 Manhattan Plot

The Manhattan plot allows all SNP effects of all chromosomes per trait. In the GUI of the upper figure in **Figure 2**, check 'Manhattan Plot'. The program will produce a Manhattan plot, a Q-Q plot, and chromosome graphs for each trait column.

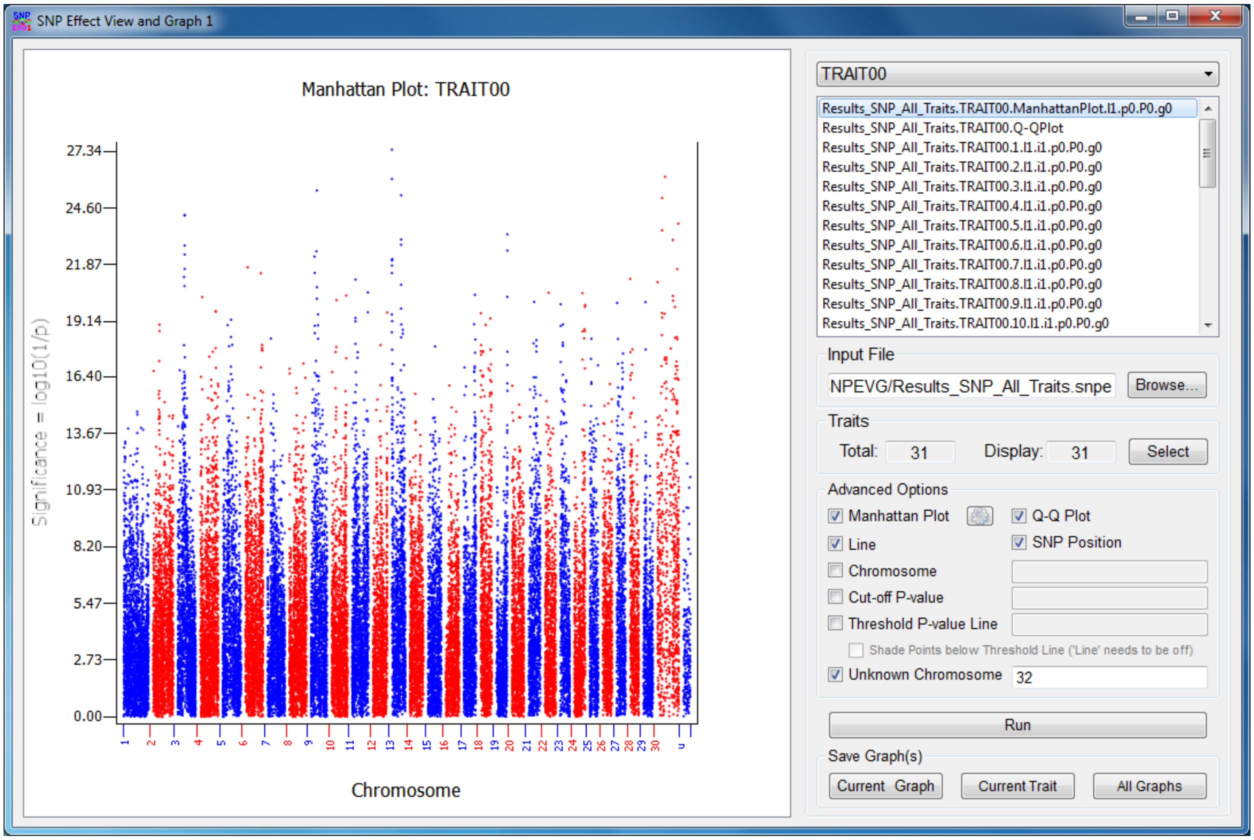


**Figure 3. Manhattan plot.**

The left window of **Figure 3** is the 'Graph window' for viewing graphs. The Manhattan plot is always the first graph in the list of each trait's graphs, and the first graph is the default display of all graphs when the user switches the trait.

The upper-right window of **Figure 3** is the 'Graph list', showing a list of graphs produced by the 'Run' button. Line 1 is the Manhattan plot, line 2 is the Q-Q plot, and the rest are chromosome

graphs of the trait. The user can switch between traits using the left or right arrow key. For example, using the right arrow key at the first line of the list, the program displays the Manhattan plot of the next trait TRAIT01. Similarly, the left arrow key displays the previous Manhattan plot for TRAIT00. The up or down arrow key allows switching between graphs in the graph list.

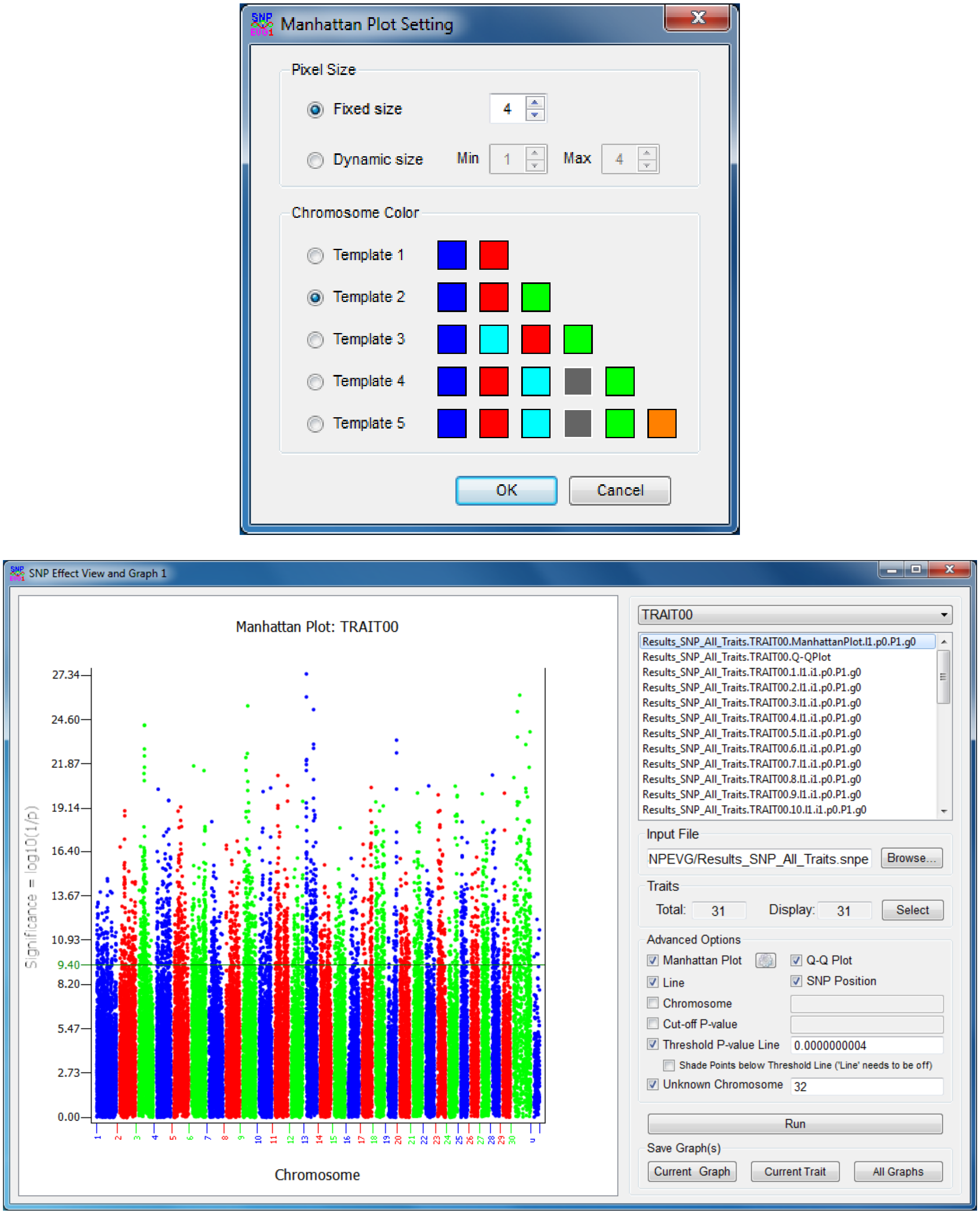


**Figure 4. Manhattan plot setting for pixel sizes and chromosome colors (upper) and Manhattan plot with threshold P-value line (lower).**

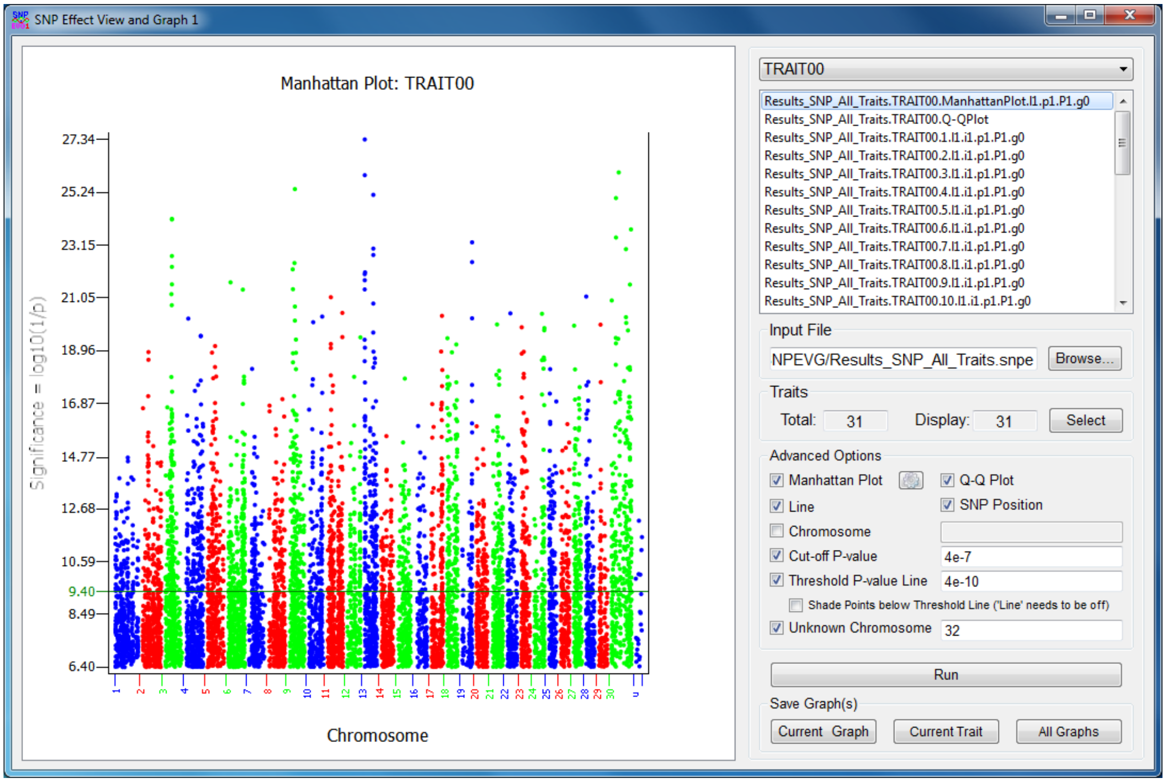


**Figure 5. Manhattan plot with threshold P-value line and without showing data points below cut-off P-value.**

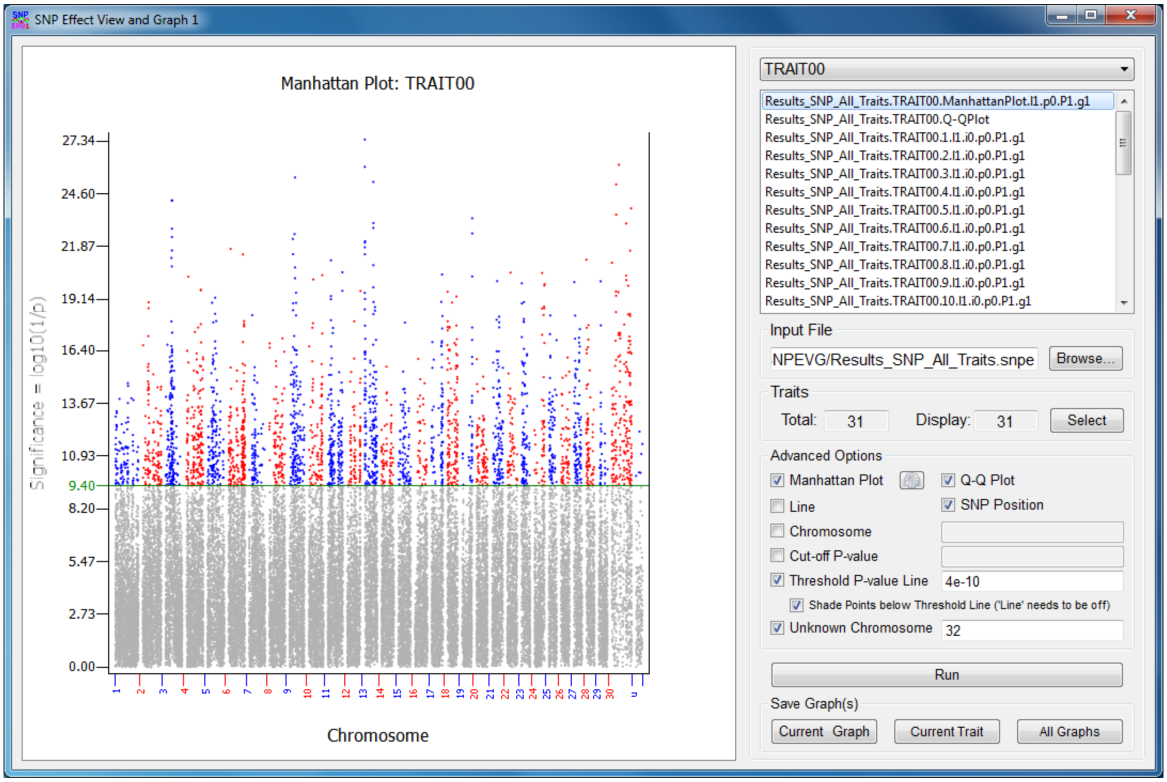


**Figure 6. Manhattan plot with shading of data points below threshold P-value line.**

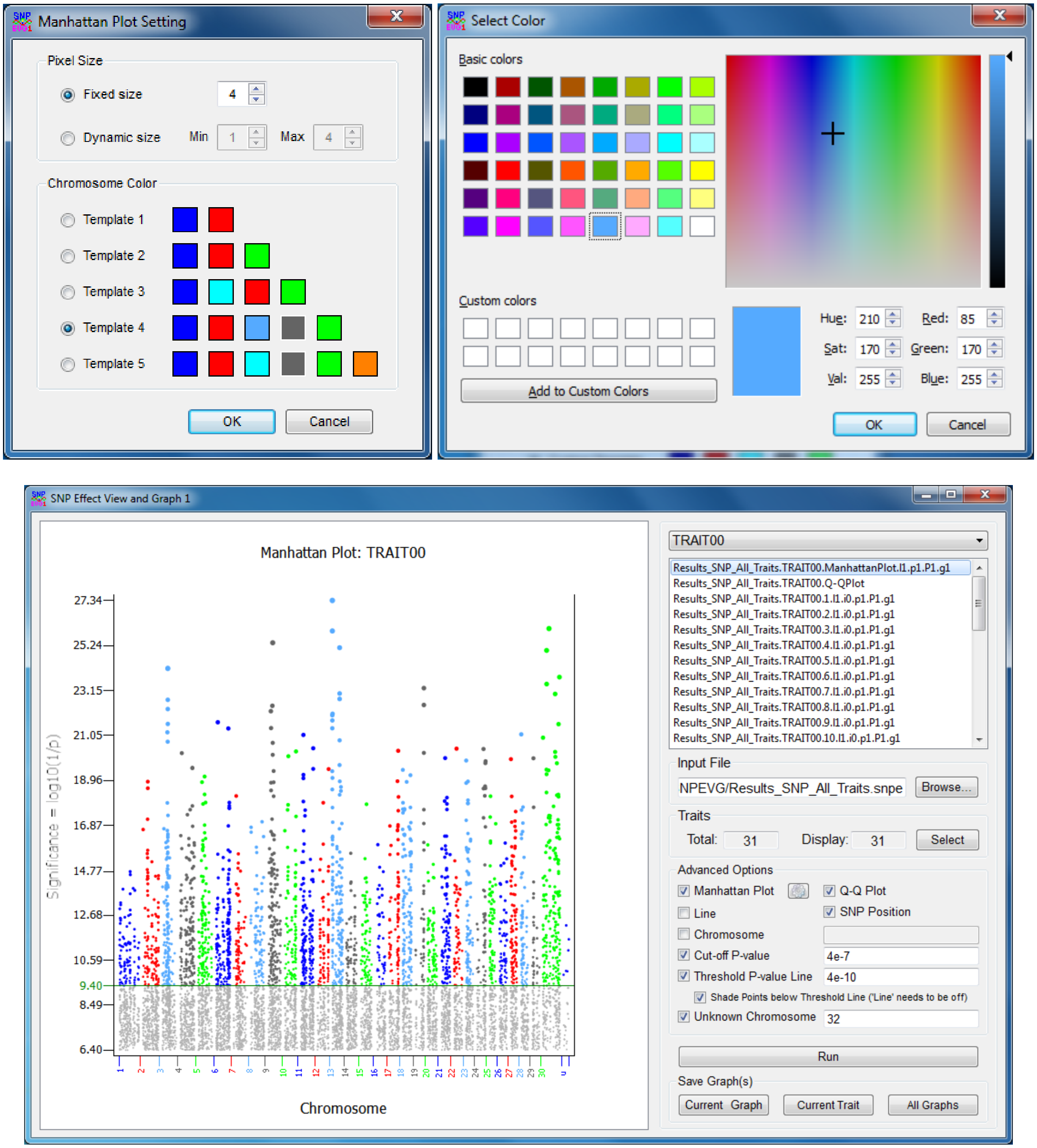


**Figure 7. Manhattan plot with user's color selection (upper) and Manhattan plot with cut-off P-value and shading of data points below threshold P-value line (lower).**

In the Manhattan plot, each chromosome width is the true width of the chromosome defined by the starting and ending SNP marker positions. The Manhattan plot has the options to change the setting of pixel sizes and colors by clicking the icon beside its checkbox (**Figure 4 and 7**). The setting of pixel sizes has two options. The fixed pixel size allows the user to change the size of pixels between 1 and 9. The dynamic pixel size allows the pixels to be varied according to the data points' significance within the user-defined range. The color of each chromosome in Manhattan plot is assigned according to the selected color template. The template colors are

extended by rotation and can be changed by double-clicking the region of a color to select a new color or moving a color by dragging and dropping the color region in the same template. Manhattan plot also has options to mark the threshold P-value line for declaring significance (**Figure 4**), to show data points above the cut-off P-value (**Figure 5**), or to shade data points below threshold P-value line (**Figure 6 and 7**).

### 4.3 Q-Q Plot for all SNP Effects

In the GUI of the upper figure in **Figure 2**, check ‘Q-Q Plot’ and the program will produce a Q-Q plot. **Figure 8** shows the Q-Q plot using all P-values.

**Warning:** If a cut-off P-value is specified, the Q-Q plot only displays P-values above the cut-off P-value. We do not recommend this option for Q-Q plot.

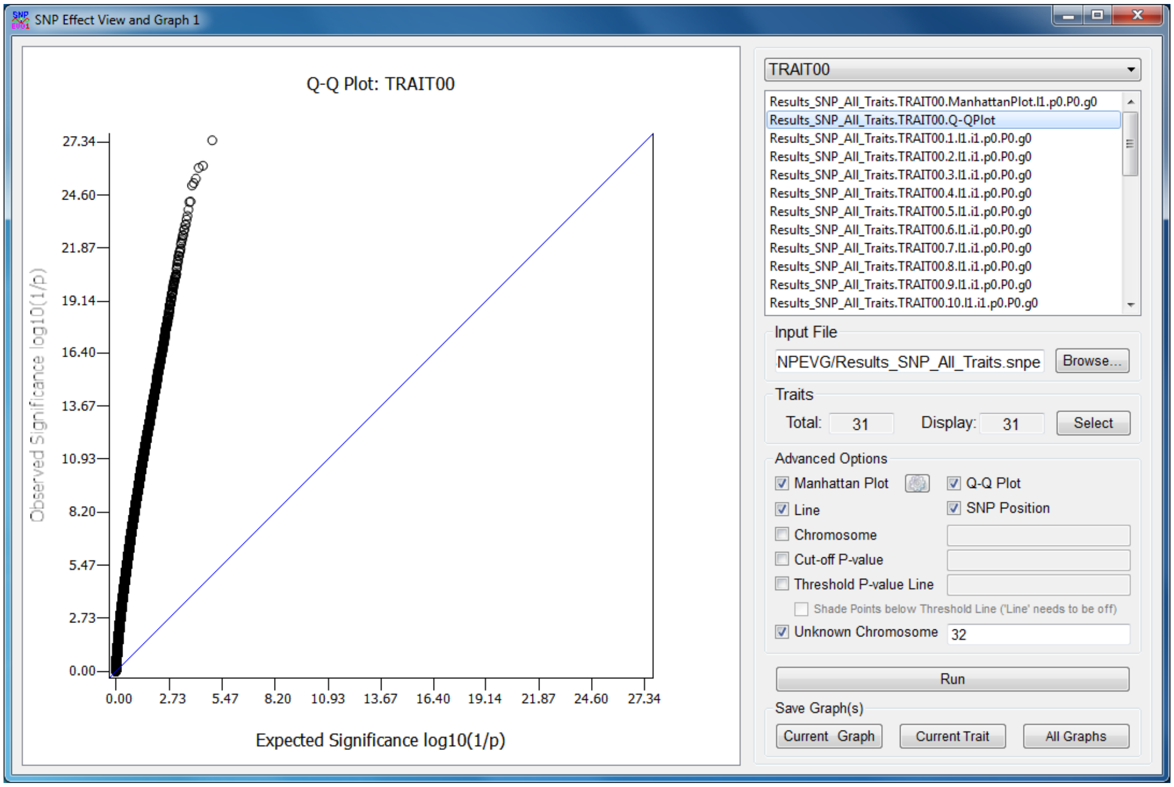


**Figure 8. Q-Q plot of all SNP effects for each trait.**

### 4.4 Chromosome Graphs

Graphs may be drawn for all chromosomes (**Figure 9**) or one selected chromosome. To select a chromosome, the specified chromosome name must match the name in the input file. Each chromosome graph has the options to use or not to use lines connecting data points (**Figure 9**), to use a threshold P-value line (**Figure 10**), or display data points above the cut-off P-value as shown in the lower figure in **Figure 10**.

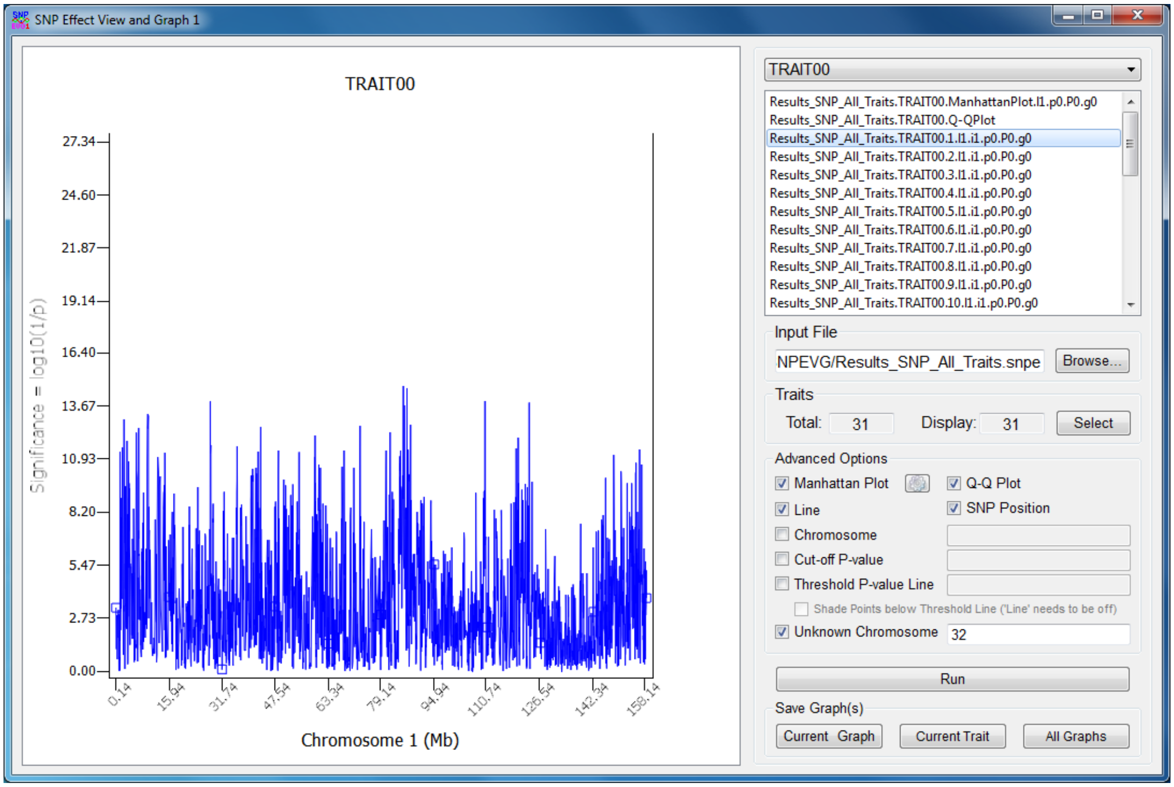

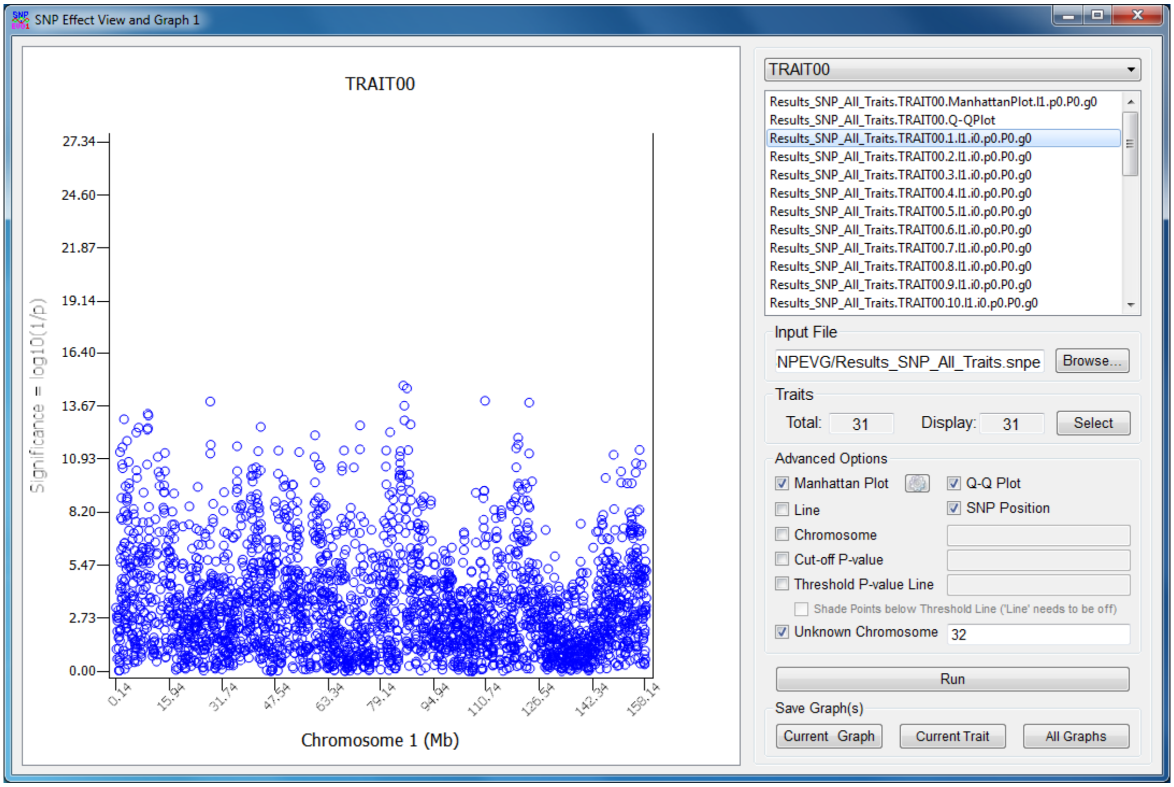

**Figure 9. Example of SNP effects by chromosome with lines (upper) and with symbols (lower).**

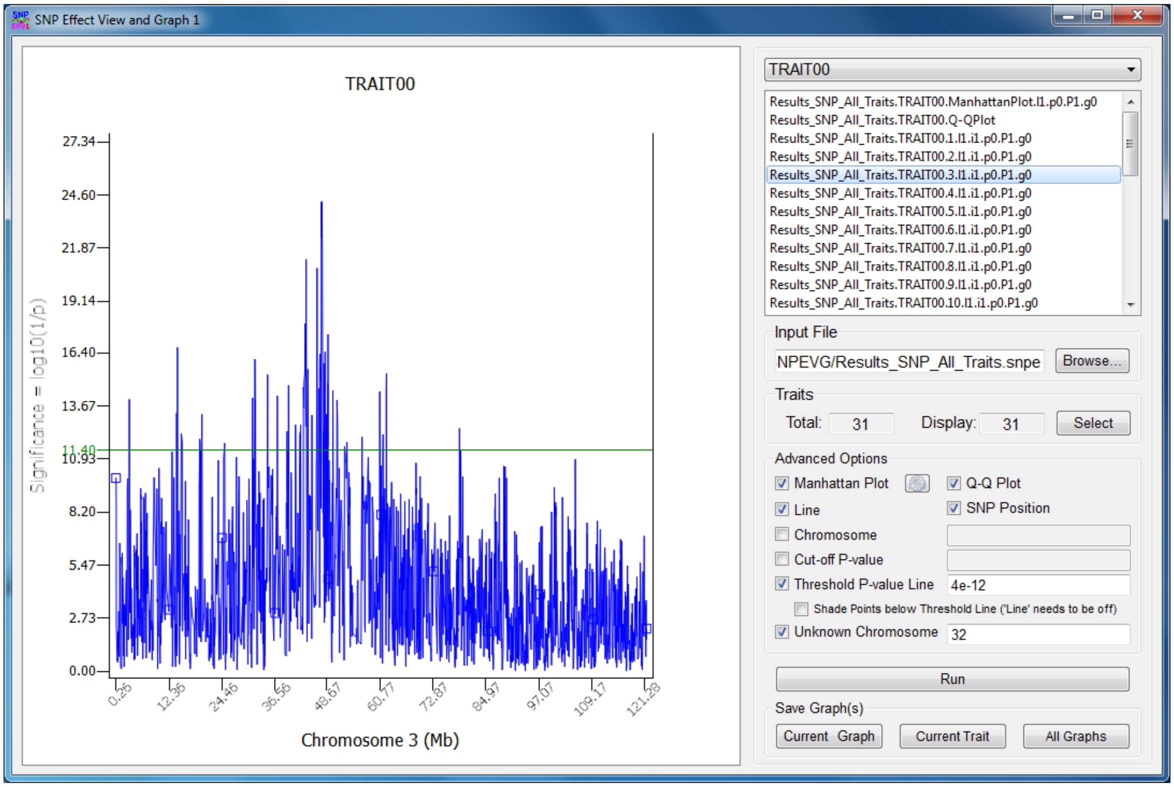


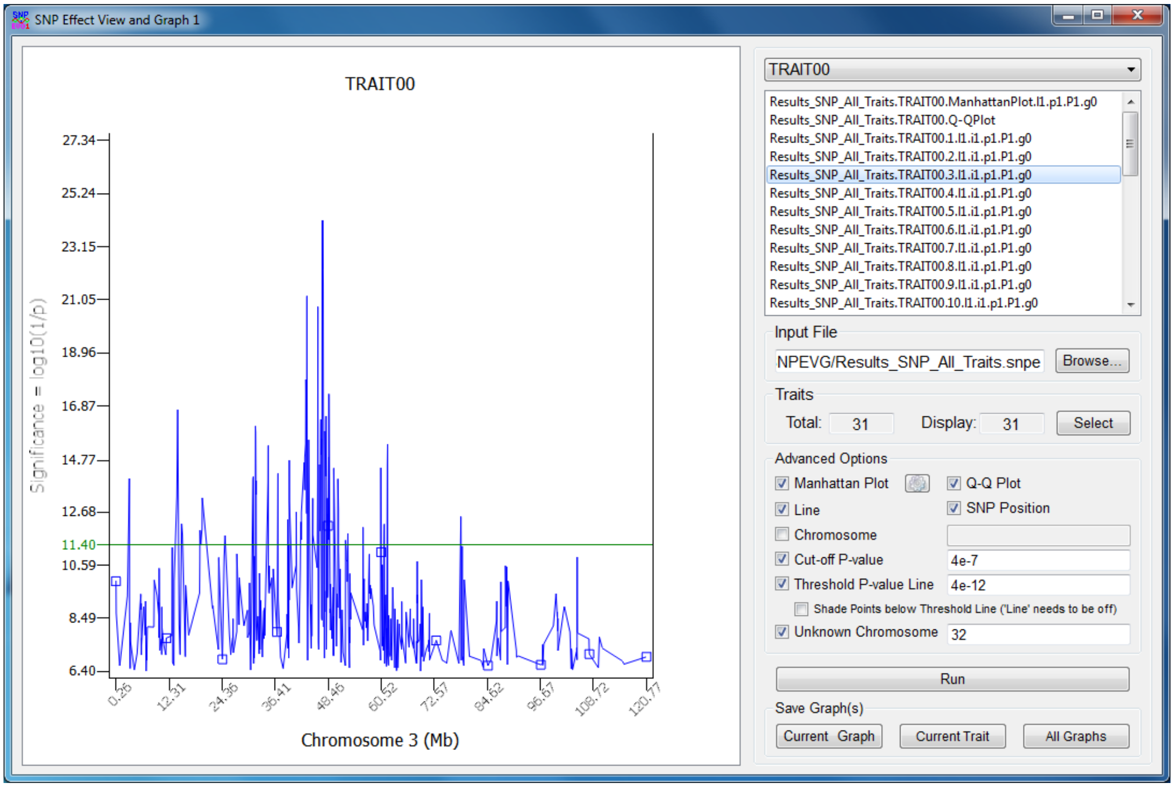


**Figure 10. Chromosome graph with threshold P-value line (upper) cut-off P-value (lower).**

### 4.5 Save Graphs to ‘png’ Files

The program saves a graph to an image in the same folder of the input file by the graph’s actual dimension. A selected graph can be saved to a ‘png’ file by clicking ‘Current Graph’, all the graphs of the currently selected trait can be saved to ‘png’ files by clicking ‘Current Trait’, and all the graphs of all traits can be saved to ‘png’ files by clicking ‘All Graphs’.

# Part II: SNPEVG2

## Multiple traits/values graph

## Any method of significance testing

## Any values of variables

## Contents


1 INTRODUCTION ....................................................................................................................16

2 INSTALLATION ....................................................................................................................17

3 INPUT FILE FORMATS ..........................................................................................................17

4 RUNNING SNPEVG2 ..............................................................................................................18

# 1 INTRODUCTION

SNPEVG2 is a graphical tool for the viewing and graphing of multiple traits on the same graph with the large quantities of genome-wide test results. The program currently uses the same input file, SNPE, of SNPEVG1 for drawing figures. Each chromosome figure can display multiple genetic effects using P-values from any methods of significance testing or original values from any methods (up to 100 effects/values). The chromosome figure also supports two axes (‘Y1’ and ‘Y2’), and the genetic effects or original values can be designated to either ‘Y1’ or ‘Y2’ axis for two groups of scales.

SNPEVG2 provides a wide-range of options for the user to choose. The GUI can produce the Manhattan plot [1], the Q-Q plot [2], and graphs for all values by chromosome by clicking one command. Any or all the graphs can be saved as png files by clicking one command. The user can increase the png resolution by using full screen. For example, the default screen size give a 667x667 png resolution and full screen gives 1119x1119 for a 1900x1200 screen resolution. The program allows users to select values from different effects or test methods for graphing and viewing. The user also has the options to draw graphs using lines or symbols, or specify a cut-off value to be shown on each graph or threshold value line to be included in the graphs. The main features and options of SNPEVG2 can be summarized as follows.

- Instant graphics of large quantities of genome-wide significance testing results or original values from any selected significance effects and original values.
- Support of ‘Y1’ and ‘Y2’ axes and selection of values for either axis.
- Immediate viewing of all graphics within the SNPEVG2 GUI.
- Scalable SNPEVG2 GUI allowing efficient and flexible use of computer screen.
- Graphics of Manhattan plot, Q-Q plot, and figures for all chromosomes and all effects/values (up to 100 effects/values) by one click of ‘run’.
- Save of one graph or all graphs for all Manhattan plots, Q-Q plots, and chromosomes by one of ‘Current Graph’ or ‘All Graphs’.
- Each Manhattan plot has the following options and features:
    - Each chromosome width is the true width of the chromosome defined by the starting and ending SNP marker positions;
    - Adjustable fixed pixel size and dynamic pixel size;
    - Color templates with the capability of custom colors;
    - Horizontal line marking the threshold value for declaring significance;
    - Display of values above the specified cut-off value;
    - Shading values below the threshold value line.
- Each chromosomal figure of all values has the following options:
    - Selection of values to be included in the figure;
    - One or two groups of values for ‘Y1’ or ‘Y2’ axes;
    - log(1/p) vs. ‘original value’;
    - Horizontal line marking the threshold value for declaring significance;
    - Display of values above the specified cut-off value;
    - Shading values below the threshold value line;
    - Optional use of lines to connect data points;
    - User specified unknown chromosome that will plot values in the sequential order of SNP markers rather than chromosome positions.
- Q-Q plot for each significant effect displaying all data points.

# 2 INSTALLATION

## 2.1 Windows x86 / x64

The installation of SNPEVG2 only requires to create a folder, to place all files in SNPEVG.zip in that folder, and then change SNPEVG2_exe file to SNPEVG2.exe file. The Windows x64 64-bit version is available by request.

## 2.2 Mac OS X 10.6 or newer 64-bit

The installation of SNPEVG2 only requires to unzip SNPEVG2_Mac.zip file to obtain SNPEVG2.app file.

# 3 INPUT FILE FORMATS

One input file with the extension 'snpe' for p-values or original values is required. The format of the input file is:

Column 1: SNP name
Column 2: chromosome number
Column 3: SNP position on the chromosome
Columns 4 through Column (n+3): 'value columns' of P-values or original values
for total n effects and values, where $0 < n \leq 100$.

As an example, the following is an input file named 'SNPEVG2_input1.snpe':

```
SNPID   Chr  Position  T1-geno    T1-add     T1-dom     T2-geno    T2-add     T2-dom
SNP01    1   571340    2.48E-03   1.11E-03   1.58E-03   2.74E-02   2.65E-01   1.11E-03
SNP02    1   845494    7.02E-04   9.07E-04   8.91E-05   2.56E-02   8.02E-02   9.07E-04
SNP03    1   883895    1.87E-02   1.71E-01   3.07E-03   1.07E-02   2.81E-03   1.71E-01
SNP04    1   929617    1.79E-03   1.57E-02   1.39E-05   9.03E-02   2.99E-04   1.57E-02
SNP05    1   950841    1.20E-03   7.07E-03   1.32E-05   8.46E-02   1.71E-03   7.07E-03
```

In the above file, column 1 is the marker name, column 2 is the chromosome number, column 3 is the marker position in units of base pairs (bp) or genetic distance in centi-Morgans, and column 4 through column 9 are P-values of genotypic effect (geno), additive effect (add) and dominance effect (dom) for each trait (T1 or T2 in this example). Any type of user defined genetic effect could be included as a value column. For example, one type of genetic effect such as additive effects for all traits could be placed in one input file, as shown in the following example named 'SNPEVG2_input2.snpe':

```
SNPID   Chr  Position  T1-add     T2-add     T3-add     T4-add     T5-add
SNP01    1   571340    2.48E-03   1.11E-03   1.58E-03   2.74E-02   2.65E-01
SNP02    1   845494    7.02E-04   9.07E-04   8.91E-05   2.56E-02   8.02E-02
SNP03    1   883895    1.87E-02   1.71E-01   3.07E-03   1.07E-02   2.81E-03
SNP04    1   929617    1.79E-03   1.57E-02   1.39E-05   9.03E-02   2.99E-04
SNP05    1   950841    1.20E-03   7.07E-03   1.32E-05   8.46E-02   1.71E-03
```

SNPEVG2 supports viewing and graphing original values for general use in addition to genetic effects. In the following input file named 'SNPEVG2_input3.snpe', one type of genetic effect for 3 traits is included from column 4 through column 6, and two different original values are included in column 7 and column 8.

```
SNPID   Chr  Position  T1-add     T2-add     T3-add     G1         G2
SNP01    1   571340    2.48E-03   1.11E-03   1.58E-03   1.74E-00   6.35E+02
SNP02    1   845494    7.02E-04   9.07E-04   8.91E-05   3.52E-01   2.34E-00
SNP03    1   883895    1.87E-02   1.71E-01   3.07E-03   1.73E+02   2.81E+01
SNP04    1   929617    1.79E-03   1.57E-02   1.39E-05   7.53E-01   4.49E-04
SNP05    1   950841    1.20E-03   7.07E-03   1.32E-05   5.73E+01   1.11E+01
```

For the given first two input files, 'SNPEVG2_input1.snpe' and 'SNPEVG2_input2.snpe', SNPEVG2 plots the log10(1/p) values according to the SNP positions on each chromosome for SNP markers with known chromosome positions, or in sequential orders of the SNP markers for SNPs in the chromosome file specified as 'unknown' by the user. For the given third input file, 'SNPEVG2_input3.snpe', SNPEVG2 is capable of plotting the first three value columns, genetic effects, by log10(1/p) values and plotting the last two value columns by original values.

For each column, SNPEVG2 produces the following graphs:
● Manhattan plot with options.

For each column of genetic effects, SNPEVG2 produces the following graphs:
● Q-Q plot with one option.

For all columns of genetic effects and original values, SNPEVG2 produces the following graph:
● Graphs for one or all chromosomes with options.

# 4 RUNNING SNPEVG2

To run SNPEVG2, open the folder that holds the SNPEVG2 files and required input files. Double click 'SNPEVG2.exe' (For Mac users, double click 'SNPEVG2.app') will open the GUI of SNPEVG2 as shown in Figure 1 below (Figures in the manual are produced in Windows 7):

The GUI shown below is scalable, i.e., the GUI size can be changed by dragging the borders of the screen, so that the GUI can use the full screen or part of the screen, allowing efficient and flexible use of the computer screen.

## 4.1 Upload Input File, Select Options, and 'Run'

In the GUI of **Figure 1**, upload an input file by clicking 'Browse…', select the options, and then click 'Run' to produce the corresponding graphs. Alternatively, the user can drag the SNPE input file and drop to the input field without using browse button. This program supports maximum 100 value columns for graphics display, and the extra value columns in the input file are discarded. When the input file is specified, total number of value columns and number of selected value columns are displayed as shown in the upper figure in **Figure 2**. All 31 value columns are selected and are displayed for axis 'Y1' by default. Clicking 'Setting', a dialog in

the lower left figure in **Figure 2** pops up and allows the user to select the values that will be displayed. The user can change each value setting for the axis 'Y1' or 'Y2' by single clicking the symbol of 'Y1' or 'Y2', and the setting toggles between the symbol 'Y1' and 'Y2'. The user can also select either axis to be the log10(1/p) value or the original value and change value texts for the display of the legend. The number of total value columns and the number of selected value columns for 'Y1' or 'Y2' are displayed and updated according to the change of the setting. If the axis is specified with the log10(1/p) effect, 'l' is displayed besides the axis notation of 'Y1' or 'Y2'. If the axis is specified with the original value, 'o' is displayed instead. In addition, the symbol color for each value can be changed by double clicking the region of a color and selecting a new color from color selection dialog. The user can also drag to copy a symbol color and drop to change another symbol color.

### 4.2 Manhattan Plot

The Manhattan plot allows all SNP effects or original values of all chromosomes per value column. In the GUI of the upper figure in **Figure 2**, 'Manhattan Plot' is enabled by default. The program produces Manhattan plots and chromosome graphs for each value column.

The left window of SNPEVG2 in **Figure 3** is the 'Graph window' for viewing graphs. The Manhattan plot is always the first graph in the list of each genetic effect or original value's graphs, and the first graph is the default display of all graphs.

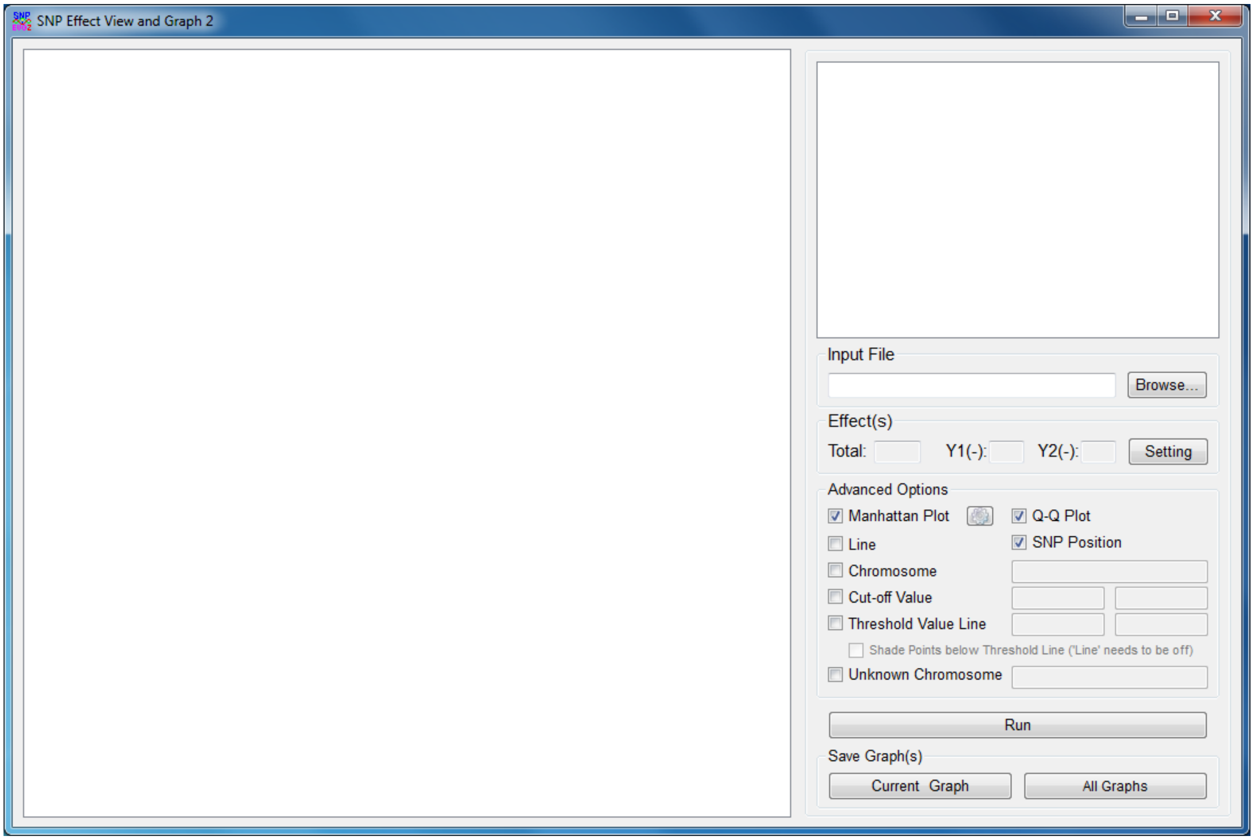


**Figure 1. Graphical User Interface (GUI) of SNPEVG2.**

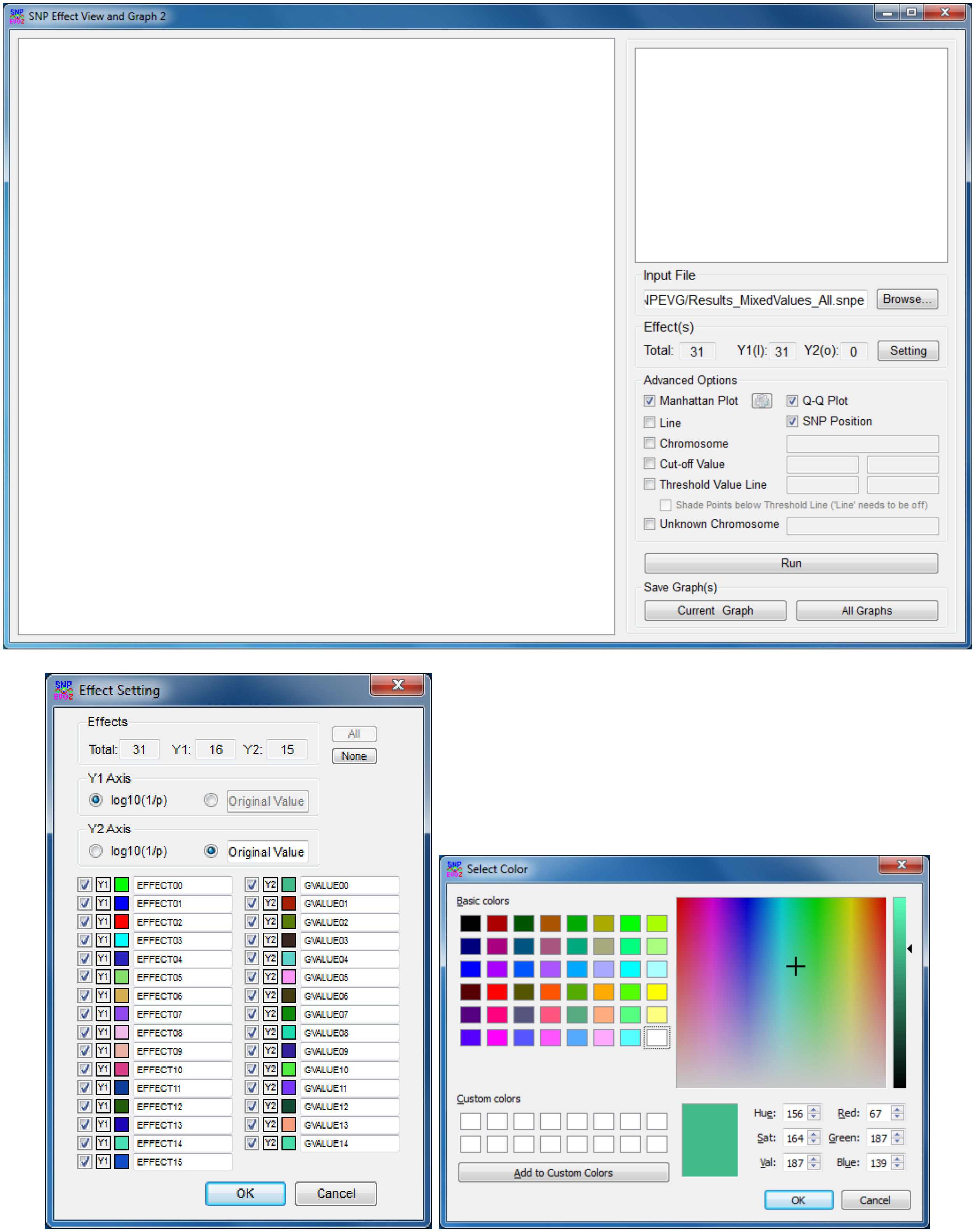


**Figure 2. Upload of an input file (upper), value and axis setting to display (lower left), and color selection dialog for values (lower right).**

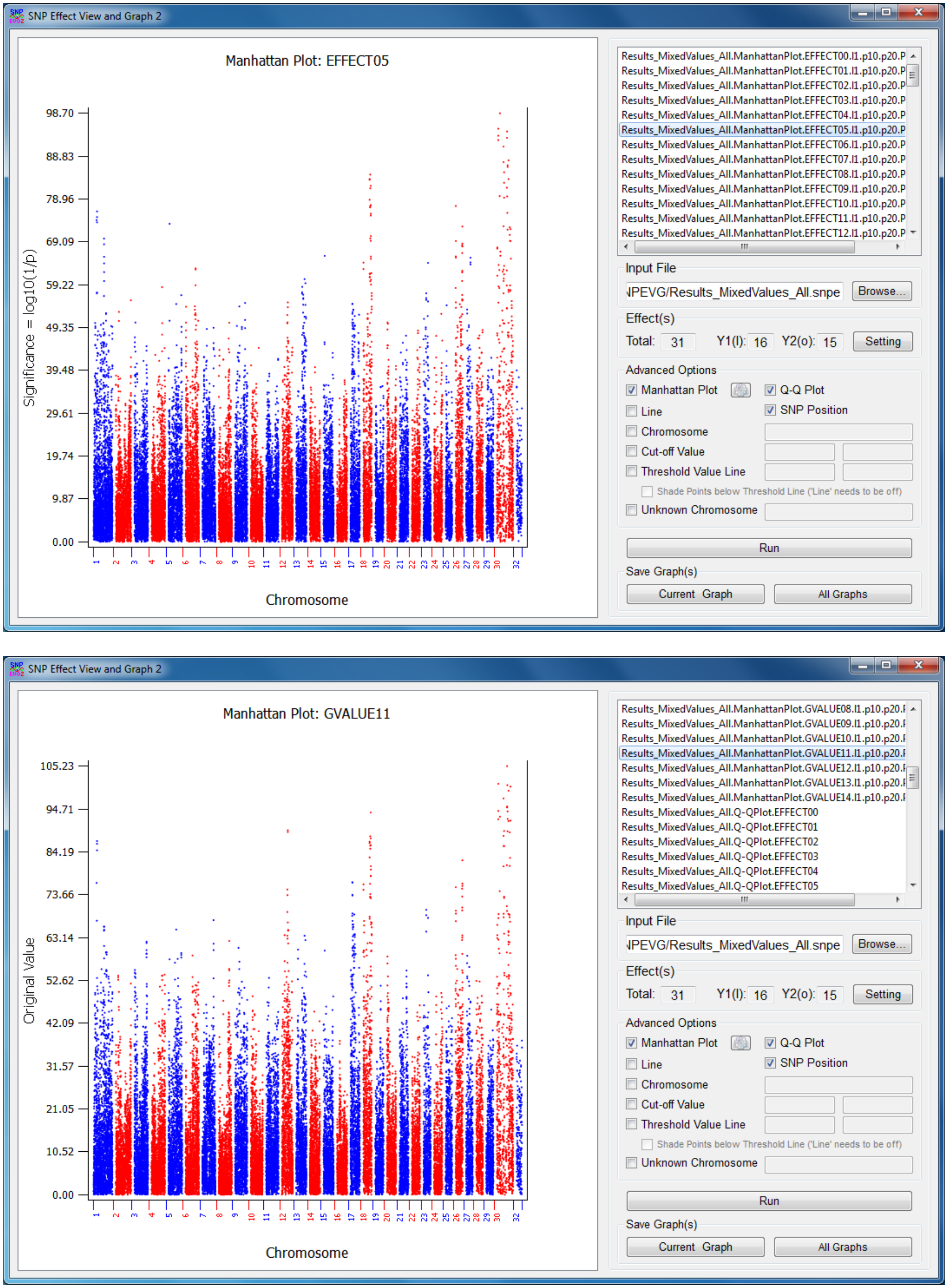


**Figure 3. Manhattan plot displaying the log10(1/p) values of genetic effects (upper) and Manhattan plot displaying original values (lower).**

The upper-right window of SNPEVG2 in **Figure 3** is the 'Graph list', showing a list of graphs produced by the 'Run' button. Manhattan plot of each genetic effect and original value is listed and is followed by the Q-Q plots and the chromosome graphs. The user can switch between graphs in the graph list by using the up or down arrow key. For example, using the down arrow key at the first line of the list, the program displays the Manhattan plot of the next genetic effect EFFECT01. Similarly, the up arrow key displays the previous Manhattan plot for EFFECT00.

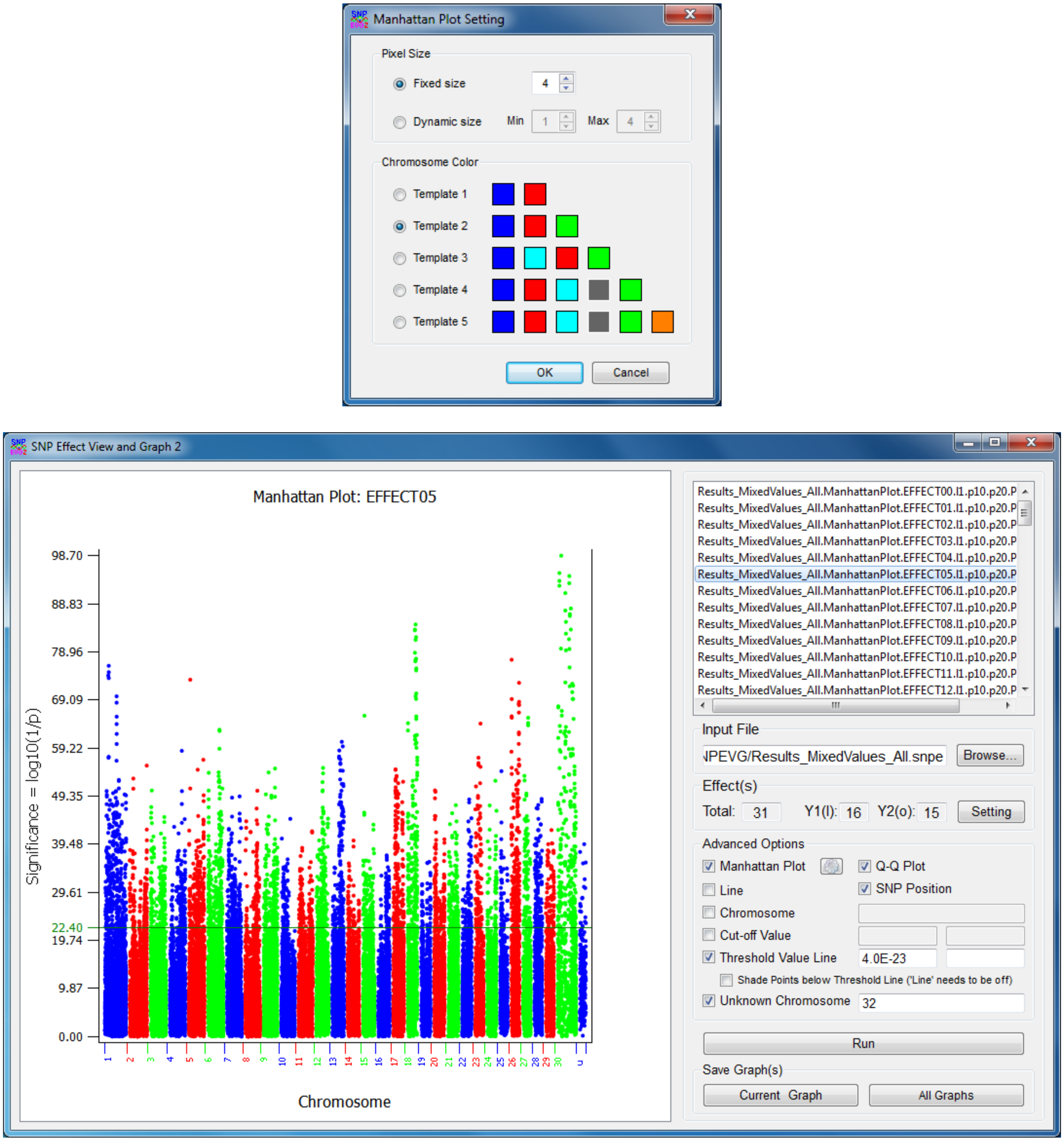


**Figure 4. Manhattan plot setting for pixel sizes and chromosome colors (upper) and Manhattan plot with threshold value line (lower).**

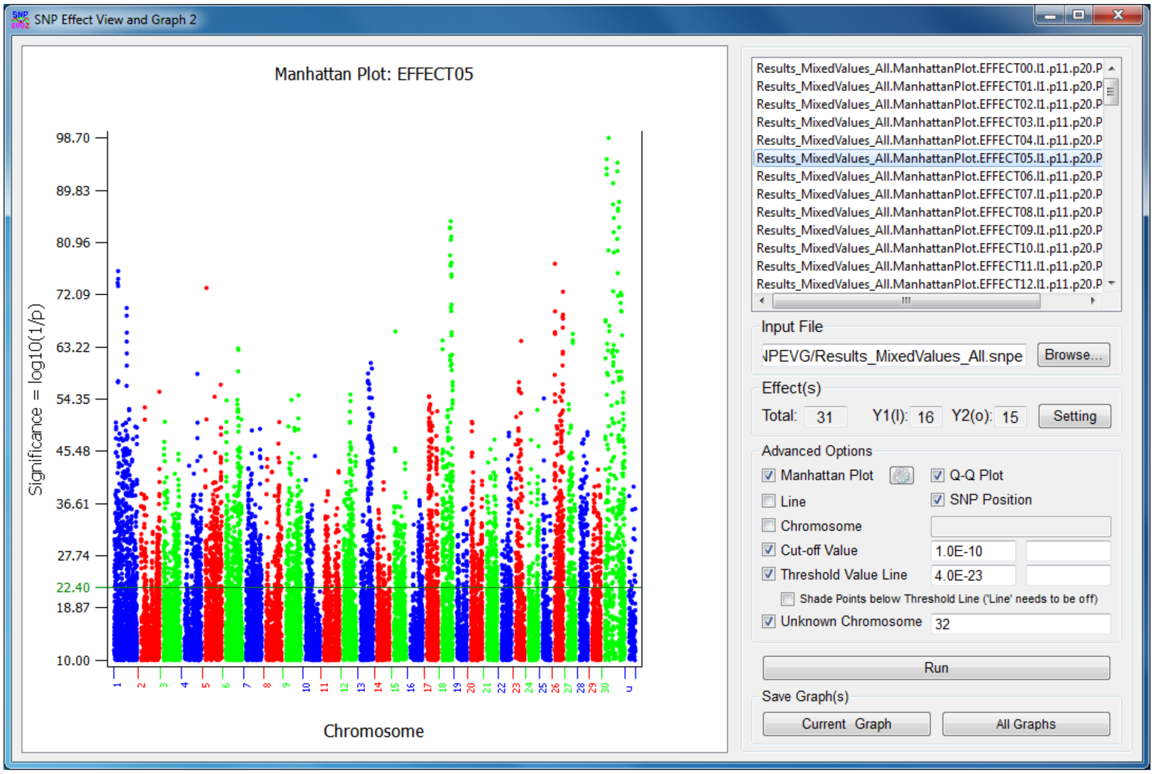


**Figure 5. Manhattan plot without showing data points below cut-off value.**

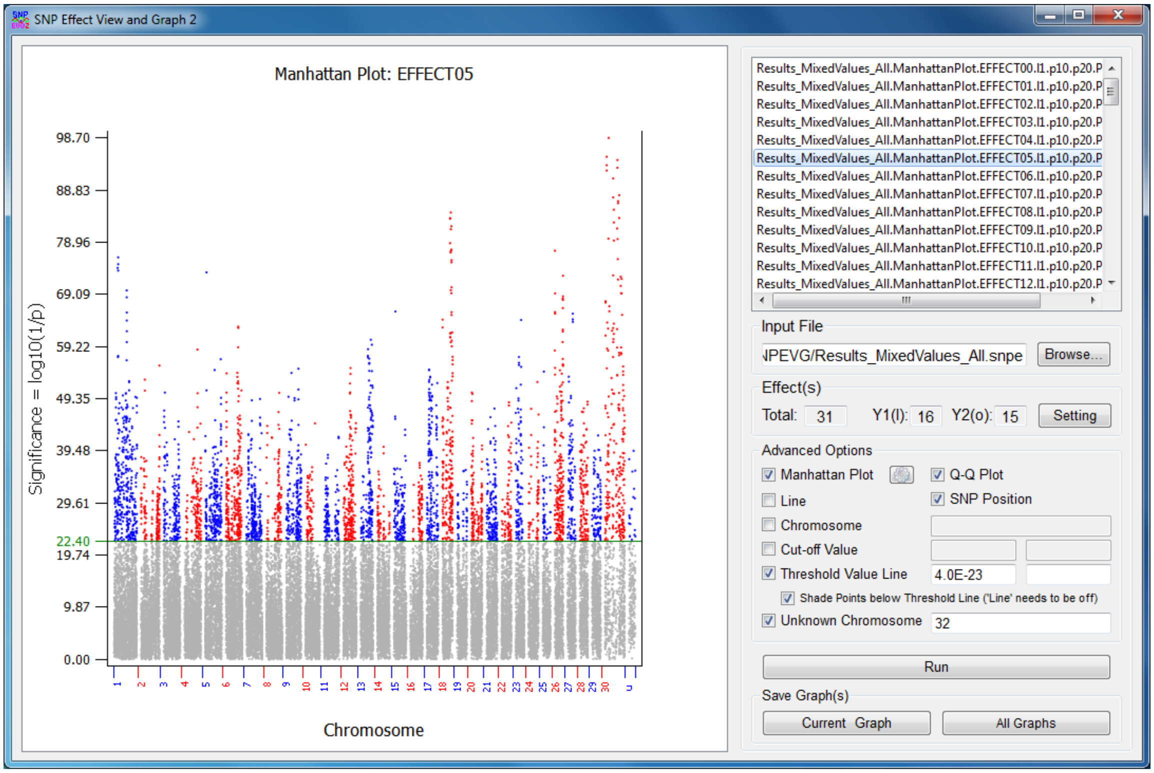


**Figure 6. Manhattan plot with shading of data points below threshold value line.**

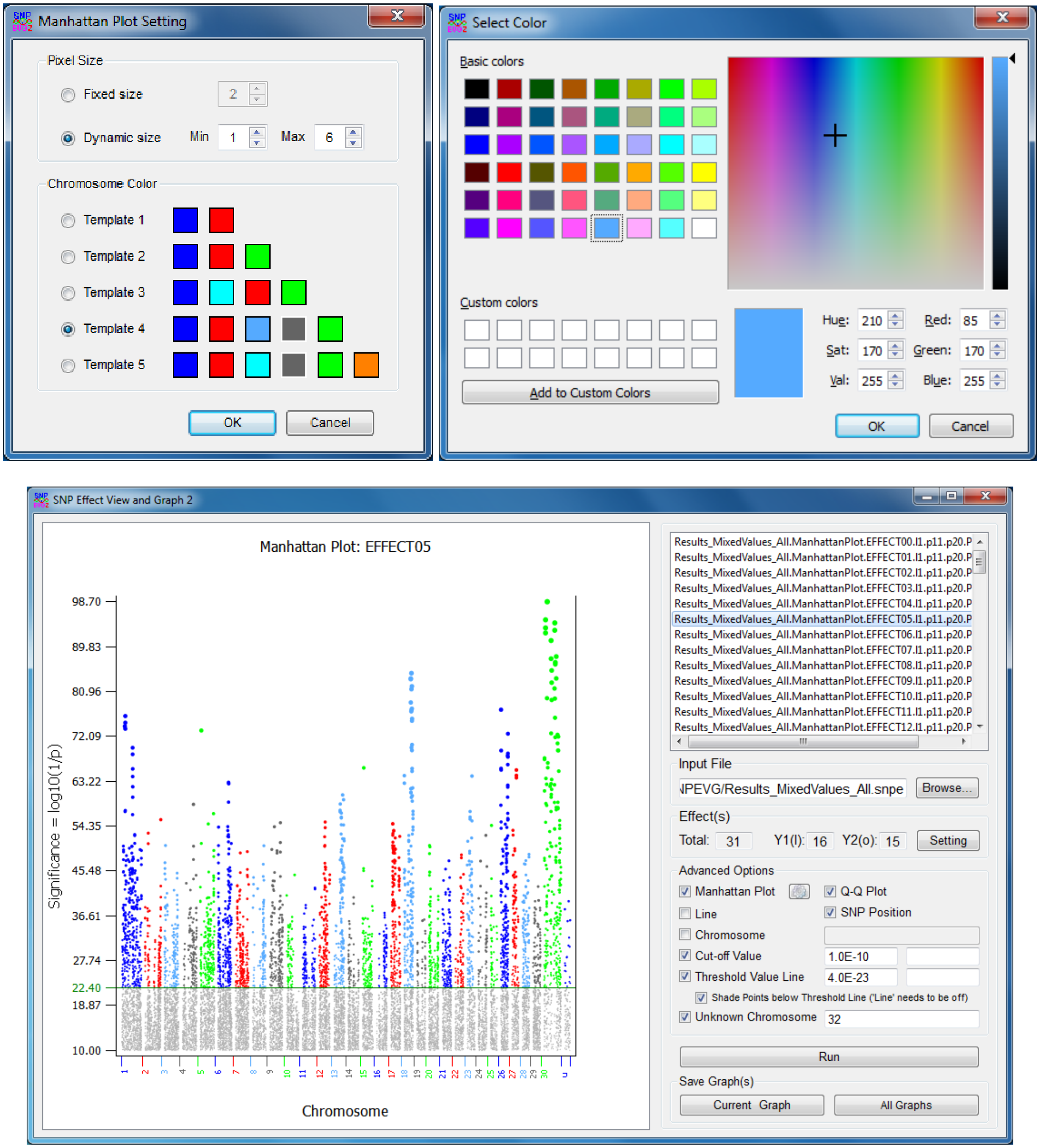


**Figure 7. Manhattan plot with user's color selection (upper) and Manhattan plot with cut-off value and shading of data points below threshold value line (lower).**

In the Manhattan plot, each chromosome width is the true width of the chromosome defined by the starting and ending SNP marker positions. The Manhattan plot has the options to change the setting of pixel sizes and colors by clicking the icon beside its checkbox (**Figure 4 and 7**). The setting of pixel sizes has two options. The fixed pixel size allows the user to change the size of pixels between 1 and 9. The dynamic pixel size allows the pixels to be varied according to the data points' significance within the user-defined range. The color of each chromosome in Manhattan plot is assigned according to the selected color template. The template colors are extended by rotation and can be changed by double-clicking the region of a color to select a new

color or moving a color by dragging and dropping the color region in the same template. Manhattan plot also has options to mark the threshold value line for declaring significance (**Figure 4**), to show data points above the cut-off value (**Figure 5**), or to shade data points below threshold value line (**Figure 6 and 7**).

### Q-Q Plot for all SNP Effects

In the GUI of the upper figure in **Figure 2**, check 'Q-Q Plot' and the program will produce a list of Q-Q plots for each genetic effect. **Figure 8** shows a Q-Q plot using all P-values.

**Warning:** If a cut-off value is specified, the Q-Q plot only displays values above the cut-off value. We do not recommend this option for Q-Q plot.

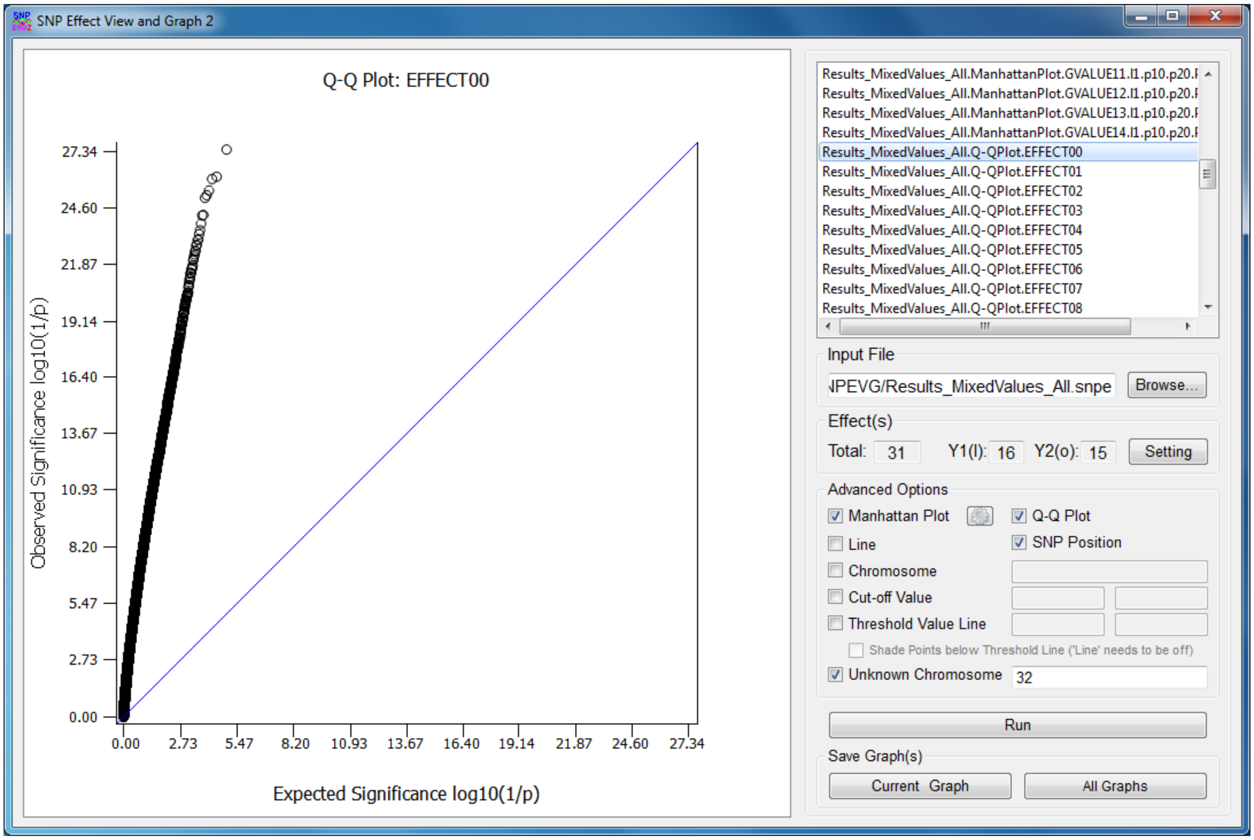


**Figure 8. Q-Q plot of all SNP effects for each genetic effect.**

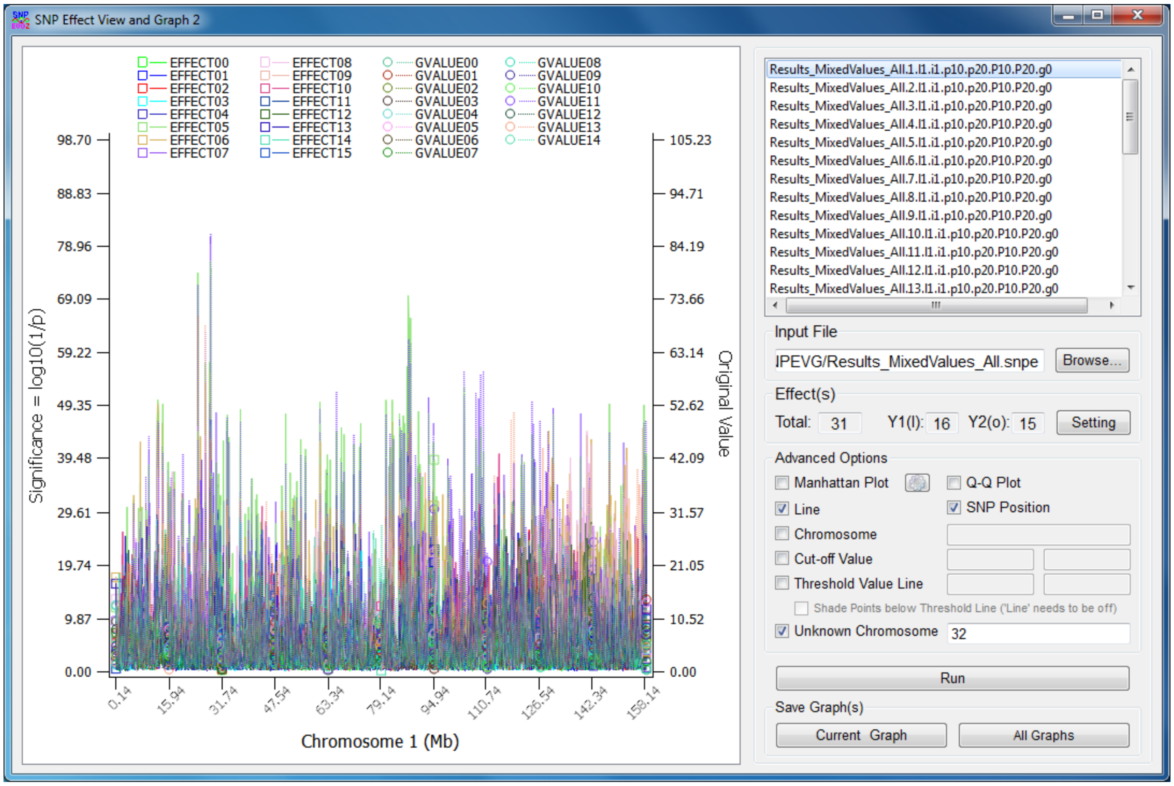

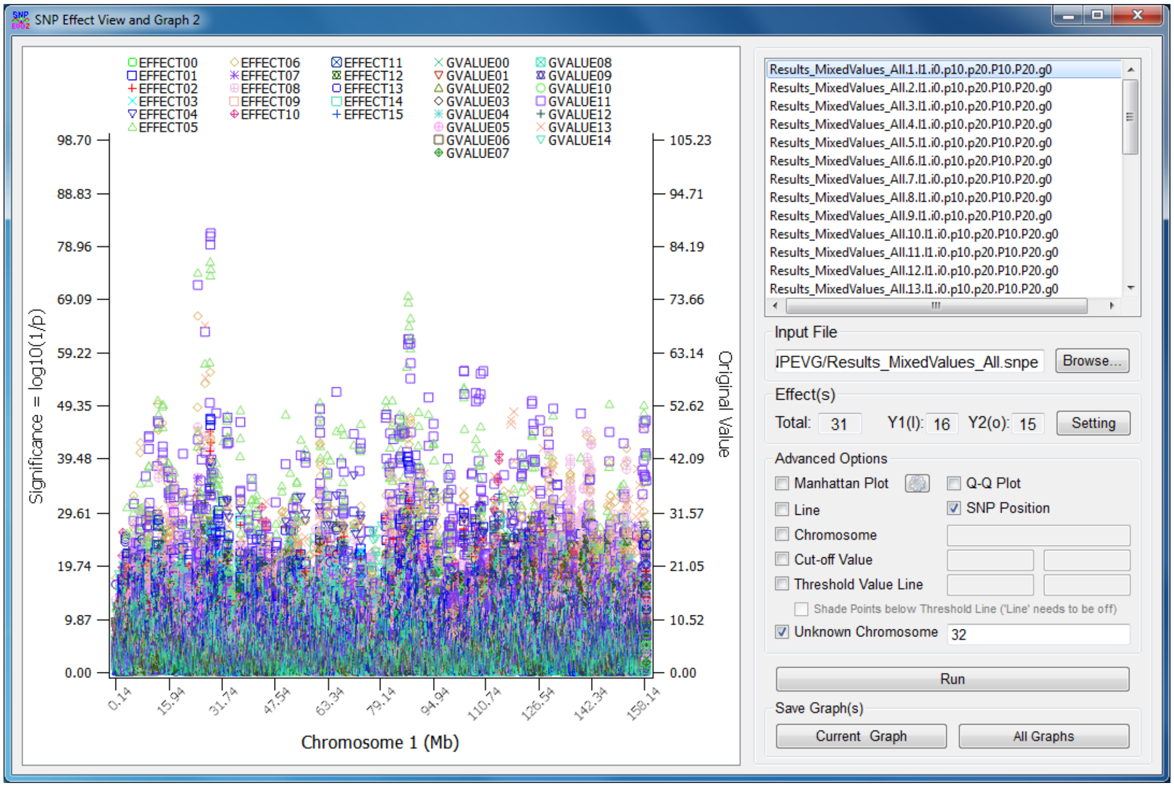

**Figure 9. Example of genetic effects and original values by chromosome with lines (upper) and with symbols (lower).**

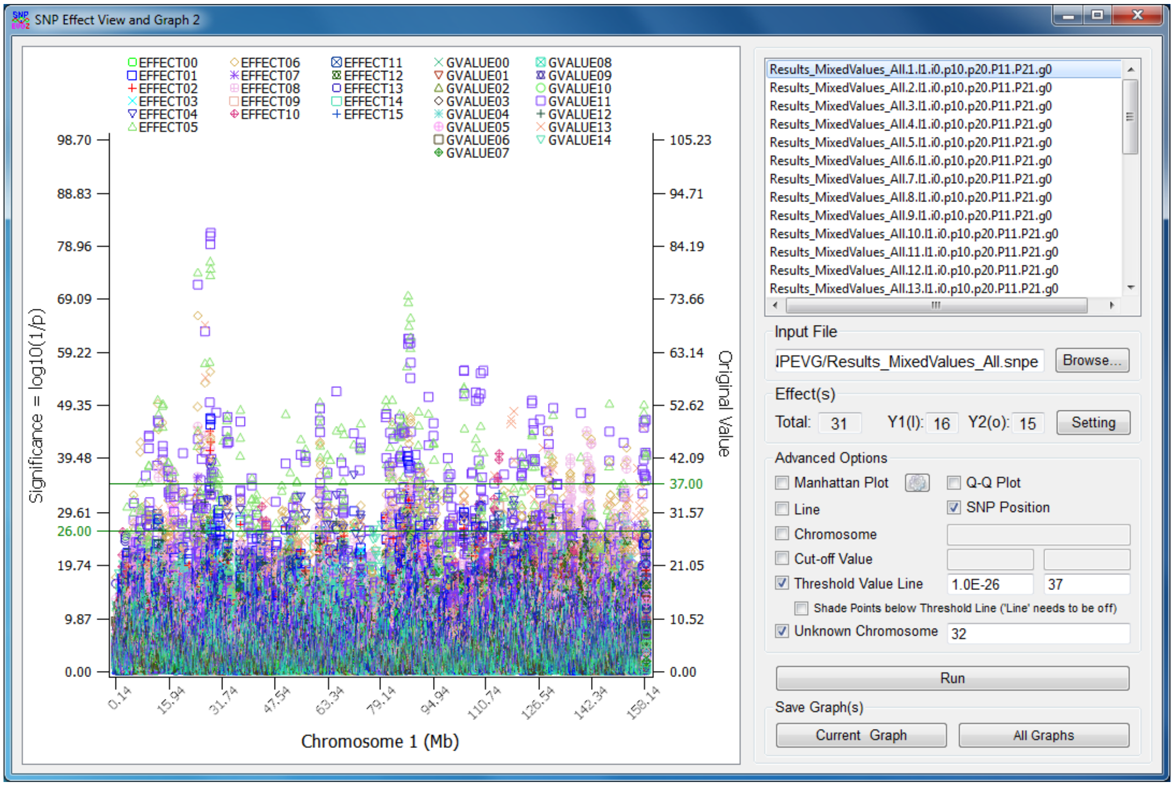


**Figure 10. Chromosome graph including threshold value lines for both axes.**

### 4.3 Chromosome Graphs

Graphs may be drawn for all chromosomes (**Figure 9**). Each chromosome graph has the options to use or not to use lines connecting data points as shown in the upper and lower figures in **Figure 9**. Genetic effects or original values can be displayed by 'Y1' or 'Y2' axis. In **Figure 10**, 16 genetic effects are displayed by 'Y1' axis using log10(1/0) value, and 15 values are displayed by 'Y2' axis using original values. The legend displays the 16 genetic effects in the left three columns and the 15 original values in the right two columns separately. The number of columns in the legend is automatically adjusted when the application window is resized. Threshold value lines for either 'Y1' or 'Y2' axis or both axes can be included (**Figure 10**). The program can display data points above the cut-off value and discard other points for chromosome 1 in the upper figure in **Figure 11** and switch to chromosome 2 by down arrow key in the lower figure in **Figure 11**. The user can display only one chromosome graph by specifying the chromosome name. To select a chromosome, the specified chromosome name must match the name in the input file. The user can select only a few values to be displayed in the effect setting dialog as shown in the upper figure in **Figure 12**. The lower figure in **Figure 12** shows that 5 effects and 2 original values are displayed for the axis 'Y1' and 'Y2' respectively. The program allows the user to copy a color between effects or values in effect setting dialog by dragging a color from one color region to another. The upper figure in **Figure 13** shows that the blue color is replicated from one effect to all others for 'Y1' axis, and the purple color is replicated for two original values of 'Y2' axis. The lower figure in **Figure 13** shows the graph of using blue color for 5 selected effects of 'Y1' axis and using purple color for 2 selected original values of 'Y2' axis.

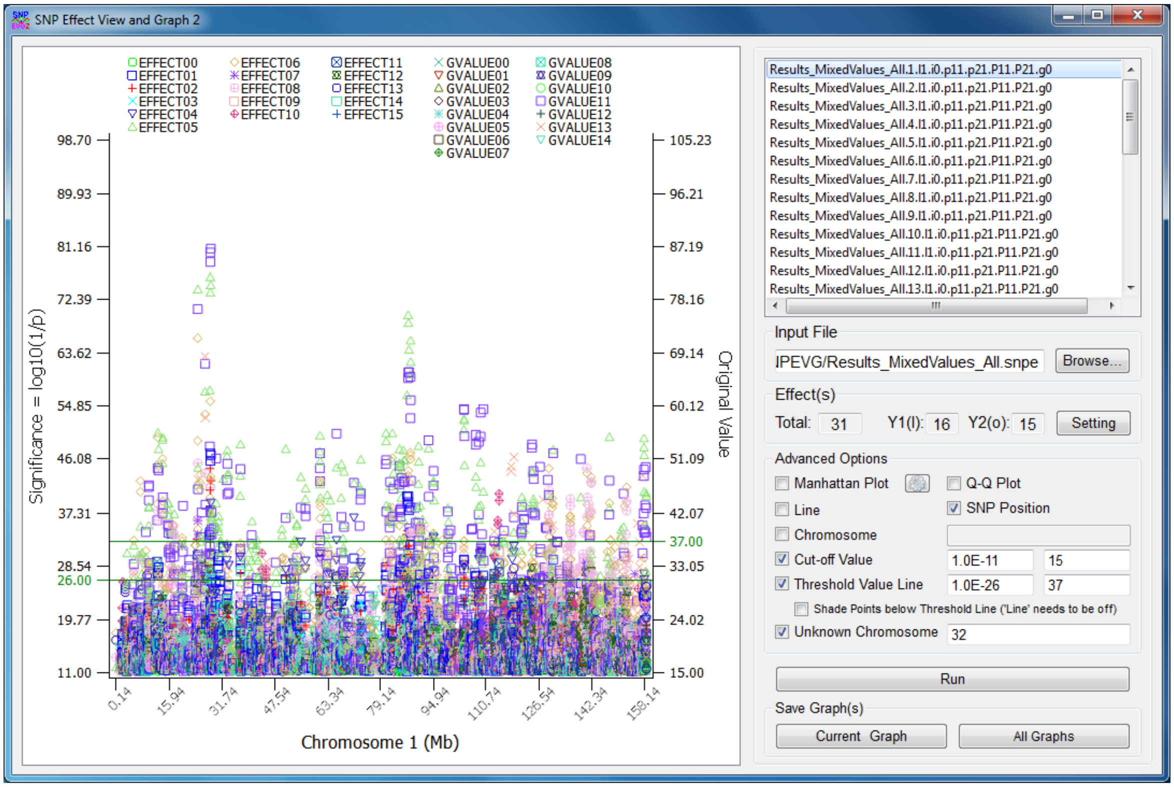


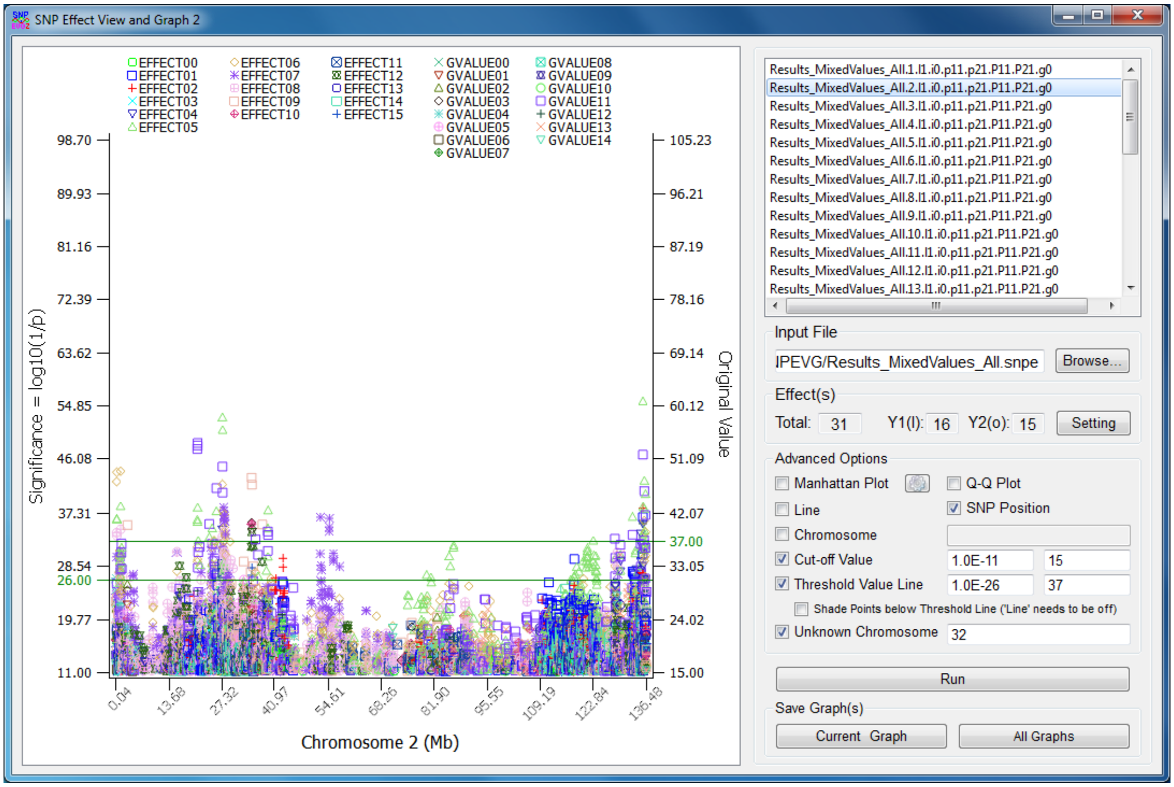


**Figure 11. Chromosome graph with threshold value lines and cut-off values for chromosome 1 (upper) and chromosome 2(lower).**

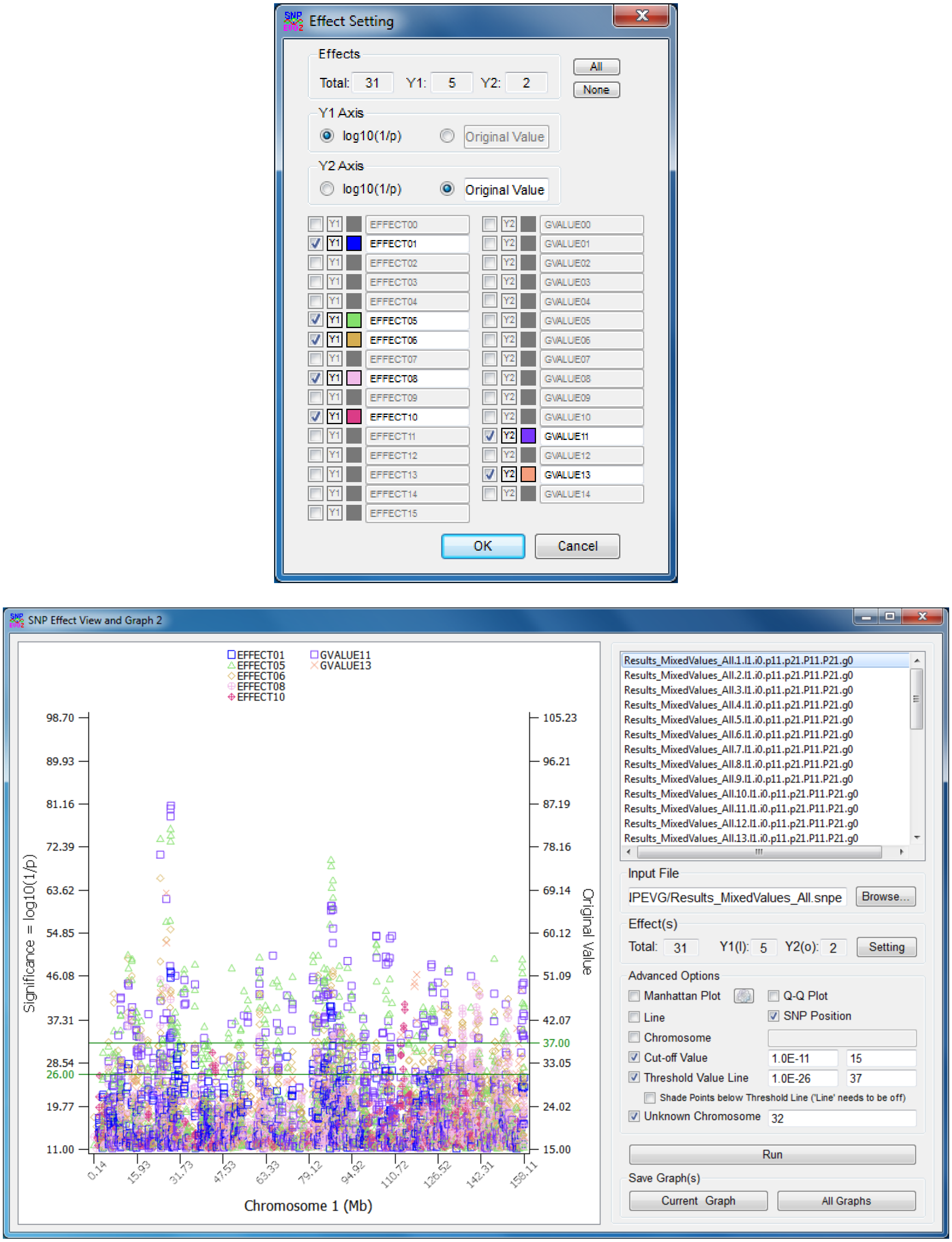


**Figure 12. Effect setting dialog for selecting only a few effects (upper) and chromosome graph with only a few effects (lower).**

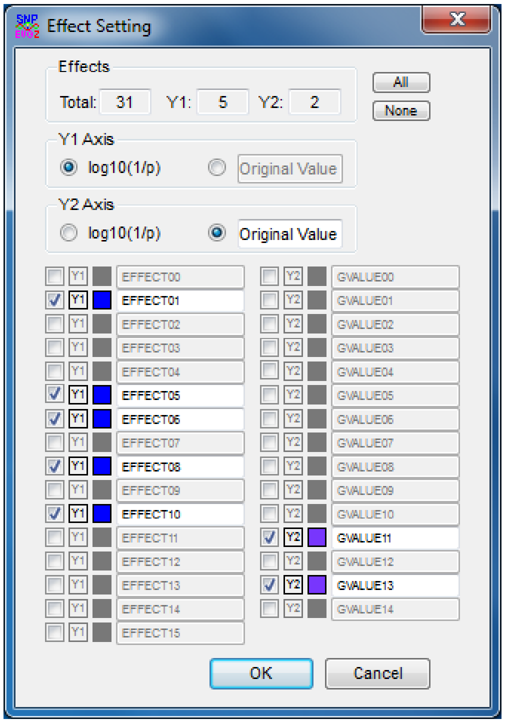


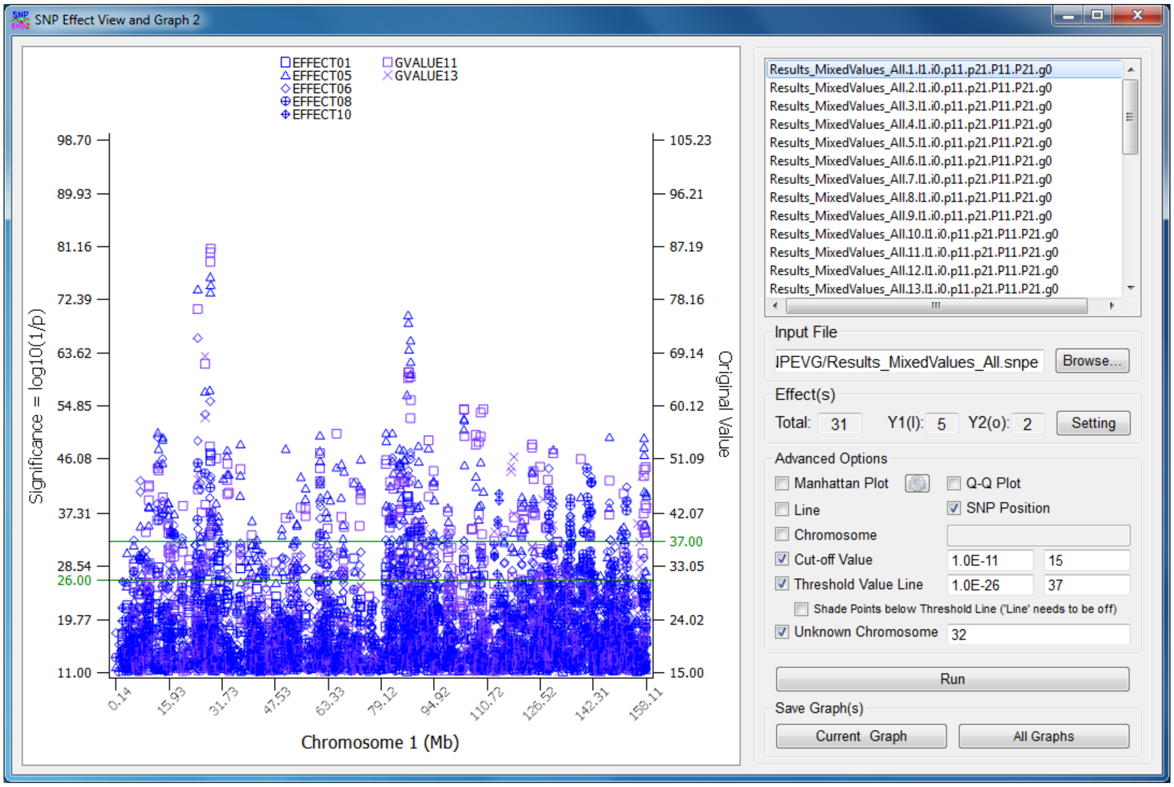


**Figure 13. Effect setting dialog for copying one effect's color to another (upper) and chromosome graph with the blue color for Y1 axis and the purple color for Y2 axis (lower).** If 'Original value' is checked, the Y-axis prints the original P-value rather than log(1/P), and the user may change 'Original value' to a user specified title.

### 4.4 Save Graphs to ‘png’ Files

The program saves a graph to an image in the same folder of the input file by the graph’s actual dimension. A selected graph can be saved to a ‘png’ file by clicking ‘Current Graph’, and all the graphs can be saved to ‘png’ files by clicking ‘All Graphs’.

# Part III: SNPEVGconvert

## Input format conversion of output file of any GWAS software to input file for SNPEVG1 and SNPEVG2

### Contents


1 INTRODUCTION ....................................................33

2 INSTALLATION ....................................................33

3 FILE FORMATS ....................................................33

4 RUNNING SNPEVGCONVERT ....................................................34

# 1 INTRODUCTION

SNPEVGconvert is a tool for input format conversion from output file of any GWAS software to the input file format for SNPEVG. The program provides the user the flexible control of file format conversion for SNPEVG1 and SNPEVG2 for the necessary data and user-selected P values.

# 2 INSTALLATION

## 2.1 Windows x86 / x64

The installation of SNPEVGconvert only requires to create a folder, to place all files in SNPEVGconvert.zip in that folder, and then change SNPEVGconvert_exe file to SNPEVGconvert.exe file for Windows x86 32-bit version or change SNPEVGconvert_x64_exe file to SNPEVGconvert_x64.exe file for Windows x64 64-bit version.

## 2.2 Mac OS X 10.6 or newer 64-bit

The installation of SNPEVGconvert only requires to unzip SNPEVGconvert_Mac.zip file to obtain SNPEVGconvert file.

# 3 FILE FORMATS

## 3.1 Parameter file (parameter.dat)

A parameter file with the name parameter.dat is required to run SNPEVGconvert. The parameter file provides user-specific controls and must have the name ‘parameter.dat’.

**Table 1: Example of the parameter file for SNPEVGconvert**

| |
|---|
| plink.assoc.linear # input file name<br>5 # number of columns in the output file<br>2 1 3 9 8 # positions of columns to print as input file for SNPEVG1 and SNPEVG2 (Must be in this order: SNP marker, chromosome, position, p-value_1 ... p-value_n)<br>assoc.snpe # output file name |

**WARNINGS:** Adding or deleting any line creates errors and is not allowed. The user may change the parameter values but may not add or delete any line in the parameter file provided by the program.

The output file of the SNPEVGconvert program is the input file of SNPEVG1 and SNPEVG2 and is the SNPE file format. The output file must be in the order of SNP marker, chromosome, position, and at least one p-value, which is determined in the parameter file.

## 3.2 Input GWAS file

The input GWAS file is determined by the user and are specified in the parameter file. This intput file must contain the SNP marker, chromosome, position, and at least one p-value for producing the output SNPE file.

# 4 RUNNING SNPEVGconvert

## 4.1 Windows

To run SNPEVGconvert, one executable file, parameter.dat, and the input GWAS file must be in the same directory. The program can be run in two ways.

Method 1

1) Open 'computer' and click the folder where the SNPEVGconvert executable file and its input files are located.
2) Double click the executable file (e.g. SNPEVGconvert.exe).

Method 2

Open the C-prompt window, and type

>chdir c:\ SNPEVGconvert_location

> SNPEVGconvert_executable_file (e.g. SNPEVGconvert.exe)

## 4.2 Mac OS X 64-bit

To run SNPEVGconvert, one executable file, parameter.dat, and the input GWAS file must be in the same directory. The program can be run in the following way.

Method 1

Open a terminal, and type

>cd / SNPEVGconvert_location

>./ SNPEVGconvert_executable_file (e.g. SNPEVGconvert)

# Part IV: SNPEVG3

## Three effects and sample size per trait

## EPISNP output file as input file

Contents

1 INTRODUCTION ....................................................36

2 INSTALLATION ....................................................36

3 FILE FORMATS ....................................................36

4 RUNNING SNPEVG3 ....................................................37

# 5 INTRODUCTION

SNPEVG3 is a graphical tool for SNP effect viewing and graphing. The program currently uses the output file of single-locus test results from the epiSNP computer package [1] as the input file for drawing figures. Each chromosome figure can display three genetic effects (genotypic, additive and dominance effects) and the number of observations. Alternatively, the user may follow the file format of the default input file for graphical viewing and drawing. For example, the user could replace the four columns of genotypic effect, additive effect, dominance effect and the number of observations by four columns of additive effects from four different traits.

# 6 INSTALLATION

## 6.1 Windows x86 / x64

The installation of SNPEVG3 only requires to create a folder, to place all files in SNPEVG.zip in that folder, and then change SNPEVG3_exe file to SNPEVG3.exe file. The Windows x64 64-bit version is available by request.

## 6.2 Mac OS X 10.6 or newer 64-bit

The installation of SNPEVG3 only requires to unzip SNPEVG3_Mac.zip file to obtain SNPEVG3.app file.

# 7 FILE FORMATS

One input file with p-values in log10(1/p) scale is required. An example is the output file from the epiSNP package named 'single_locus_fig.out'. The following is an example of this file:

```
The output for making figures(m,a,d).
Trait          Chr   Locus          M           A           D          #_Ind
trait1         14    M112           0.704E+00   0.107E+01   0.285E+00   295
trait2         14    M112           0.492E+00   0.127E+00   0.864E+00   295
trait1         14    M212           0.401E+00   0.689E-01   0.748E+00   295
trait1         19    M7             0.184E+01   0.180E+01   0.803E+00   290
trait2         19    M7             0.103E+01   0.133E+01   0.220E+00   290
trait1         19    M8             0.398E+00   0.601E+00   0.347E+00   295
trait2         19    M8             0.188E+00   0.413E+00   0.121E+00   295
trait1         19    M9             0.763E+00   0.930E+00   0.663E+00   295
trait2         19    M9             0.105E+01   0.122E+00   0.145E+01   295
trait1         19    M10            0.170E+01   0.174E+01   0.967E+00   292
trait2         19    M10            0.782E+00   0.842E+00   0.697E+00   292
trait1         19    M11            0.692E+00   0.345E+00   0.940E+00   295
trait2         19    M11            0.473E+00   0.113E+00   0.841E+00   295
trait1         19    M12            0.396E+00   0.521E+00   0.434E+00   295
trait2         19    M12            0.119E+00   0.115E+00   0.309E+00   295
```

In the above file, column 1 is the trait name, column 2 is the chromosome number, column 3 is the marker name, column 4 is log10(1/p) values for marker genotypic effect, column 5 is log10(1/p) values for additive effect, column 6 is log10(1/p) values for dominance effect, and column 7 is the number of individuals with known genotypes for this marker. With this input

file, SNPEVG3 plots the log10(1/p) values in the sequential order of the markers on each chromosome.

A chromosome and position file is required if the user wants to plot the log10(1/p) values according to the markers' physical location defined either by base pair location or genetic distance in centi-Morgans. The file name's extension is 'pos'. The following is a short example of this position file:

```
SNP            Chr         Position
M12             1          135098
M7              1          267940
```

In the above file, column 1 is the marker name, column 2 is the chromosome number, column 3 is the marker position, and the first row is the header of the file. Marker name, chromosome number, and marker position start in the second row.

# 8 RUNNING SNPEVG3

To run SNPEVG3, open the folder that holds the SNPEVG3 files and required input files. Double click 'SNPEVG3.exe' (For Mac users, double click 'SNPEVG3.app') will open a graphical user interface (GUI) of SNPEVG3 as shown below (Figures in the manual are produced in Windows 7):

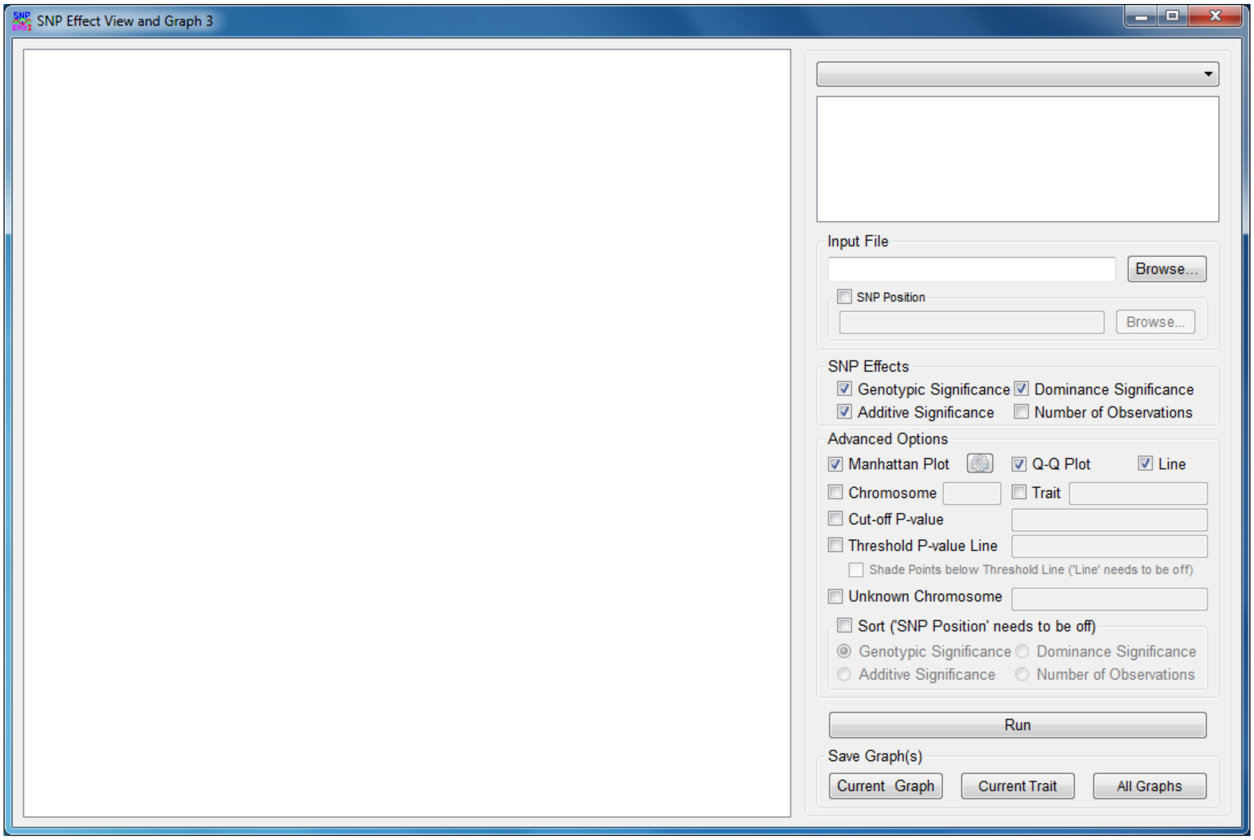


**Figure 1. Graphical User Interface of SNPEVG3.**

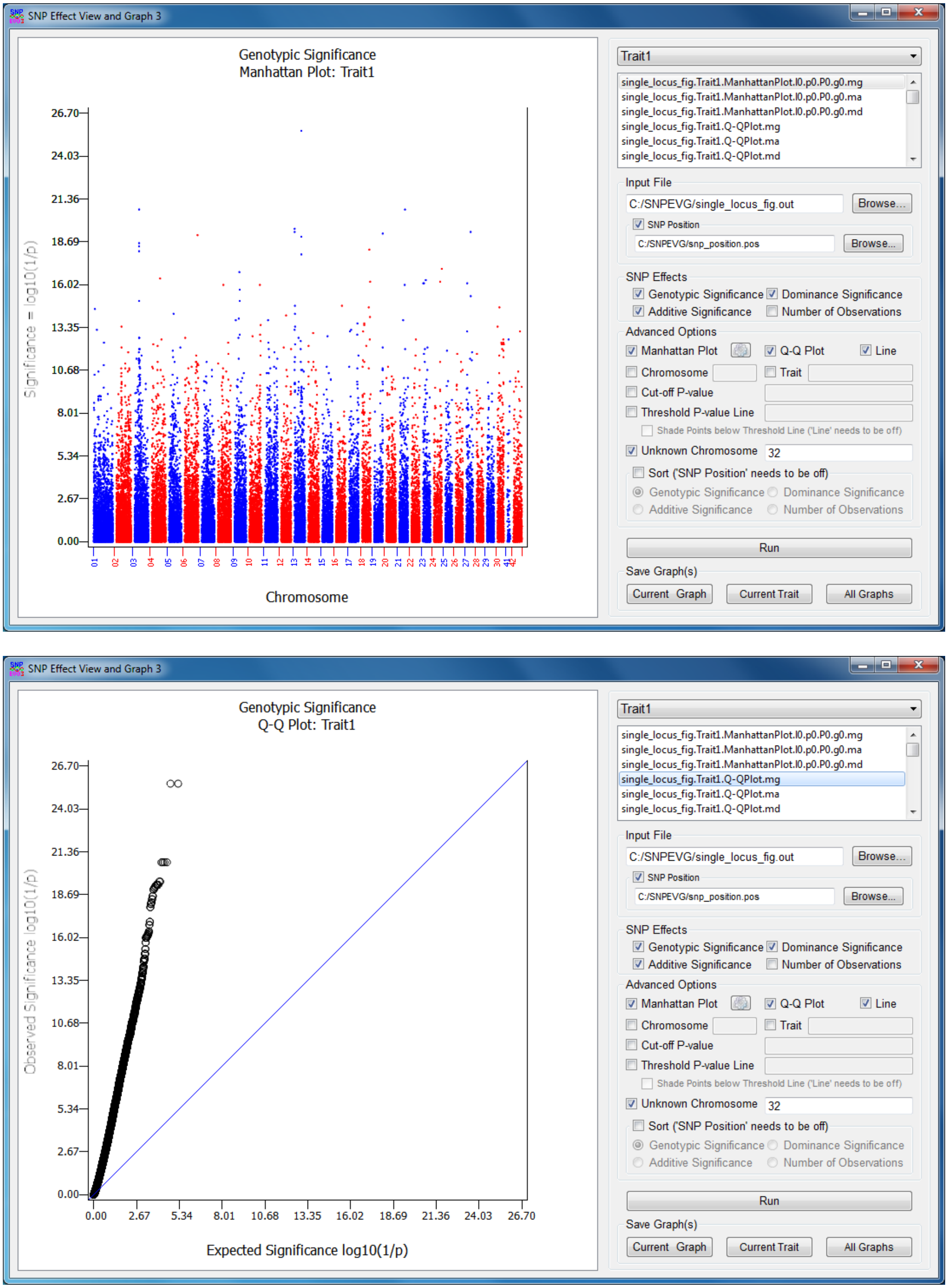


**Figure 2. Manhattan and Q-Q plots by SNPEVG3.**

In the GUI of **Figure 1**, specify the input file(s), select the options, and then click 'Run' to produce the corresponding graphs for one or multiple traits. Alternatively, the user can drag the input file and drop to the corresponding input field without using browse button.

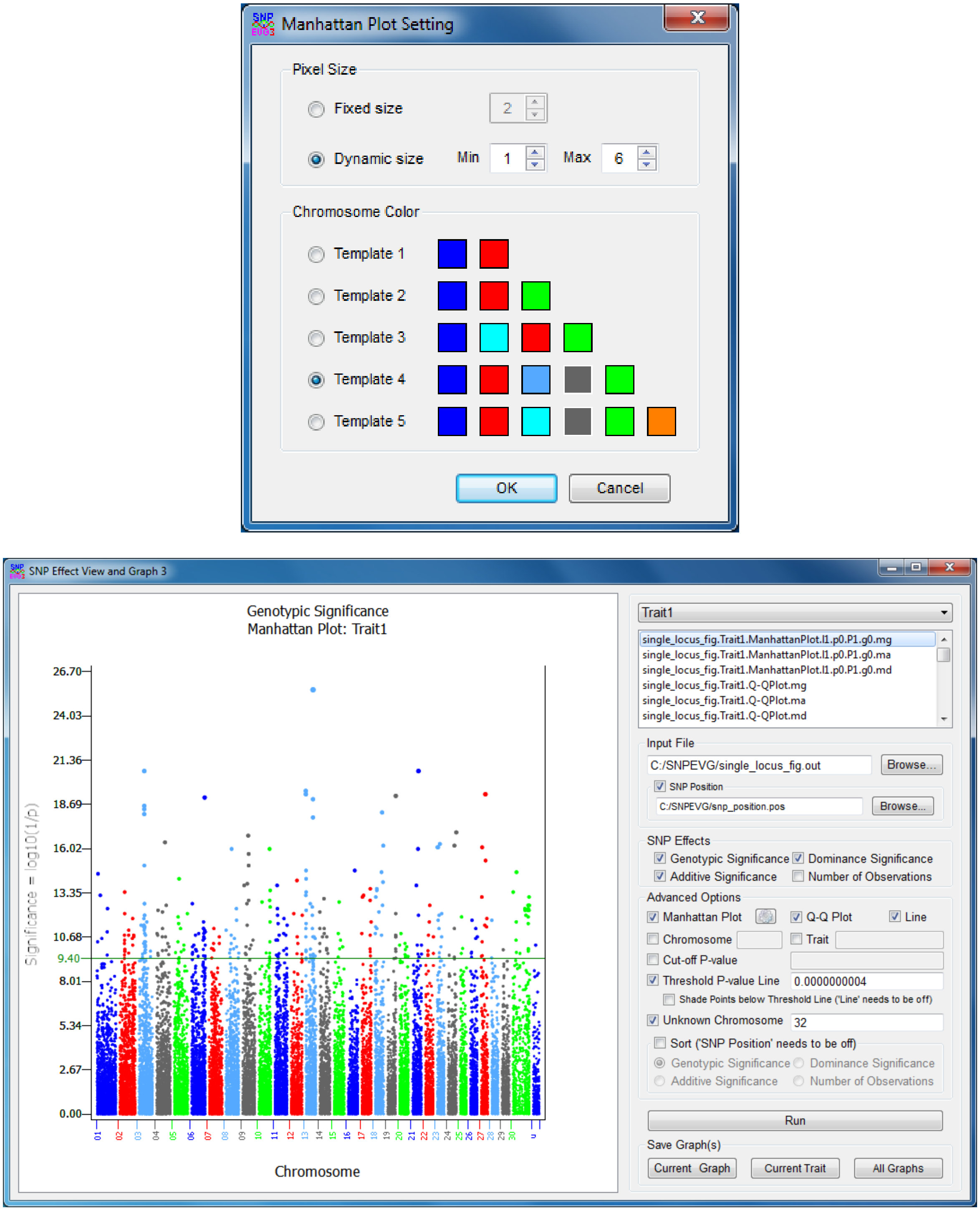


**Figure 3. Manhattan plot setting for pixel sizes and chromosome colors (upper) and Manhattan plot with threshold P-value line.**

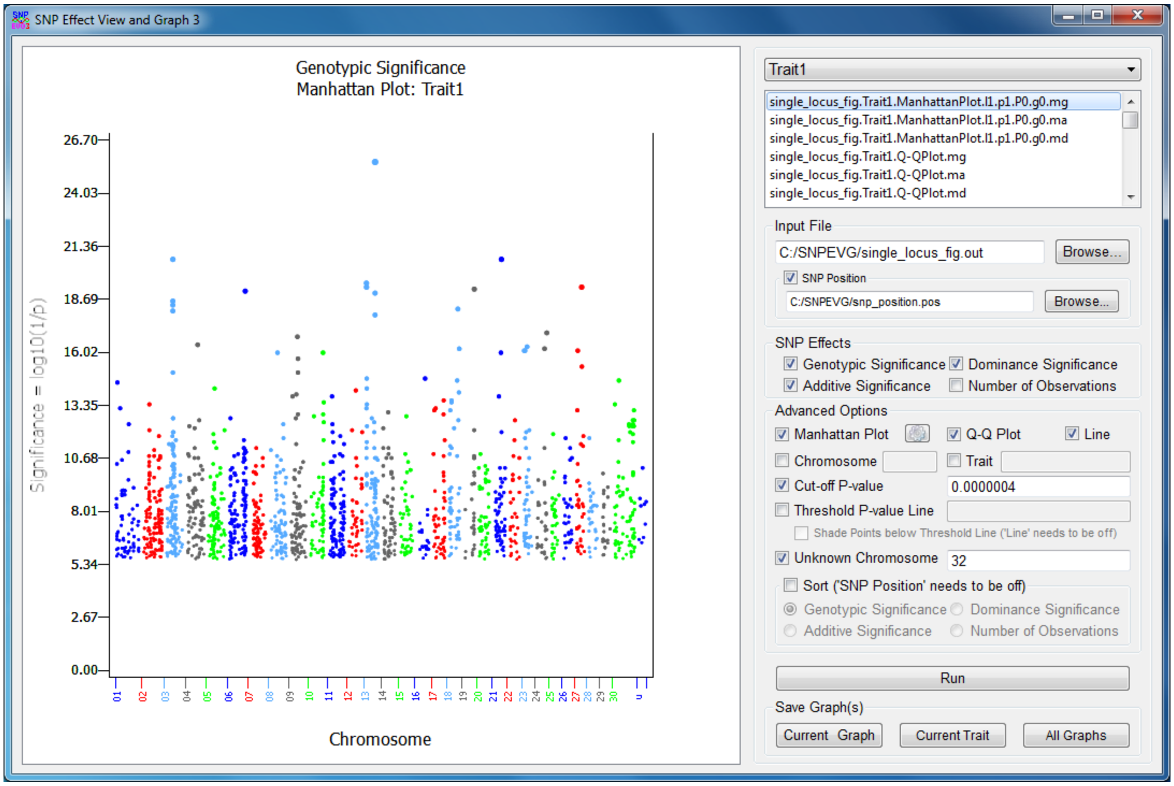


**Figure 4. Manhattan plot showing data points above cut-off P-value.**

### 4.1 Manhattan and Q-Q Plots

The Manhattan plot [2] and Q-Q plot [3] by SNPEVG3 have the same features as described for SNPEVG1 except the figure legend for the Manhattan plot (**Figure 2**). The Manhattan plot has the options to change the setting of pixel sizes and colors by clicking the icon beside its checkbox (**Figure 3**). The setting of pixel sizes has two options. The fixed pixel size allows the user to change the size of pixels between 1 and 9. The dynamic pixel size allows the pixels to be varied according to the data points' significance within the user-defined range. The color of each chromosome in Manhattan plot is assigned according to the selected color template. The template colors are extended by rotation and can be changed by double-clicking the region of a color to select a new color or moving a color by dragging and dropping the color region in the same template. Manhattan plot also has options to mark the threshold P-value line for declaring significance (**Figure 3**), to show data points above the cut-off P-value (**Figure 4**), or to shade data points below threshold P-value line (**Figure 5**).

**Warning:** If a cut-off P-value is specified, the Q-Q plot only displays P-values above the cut-off P-value. We do not recommend this option for Q-Q plot.

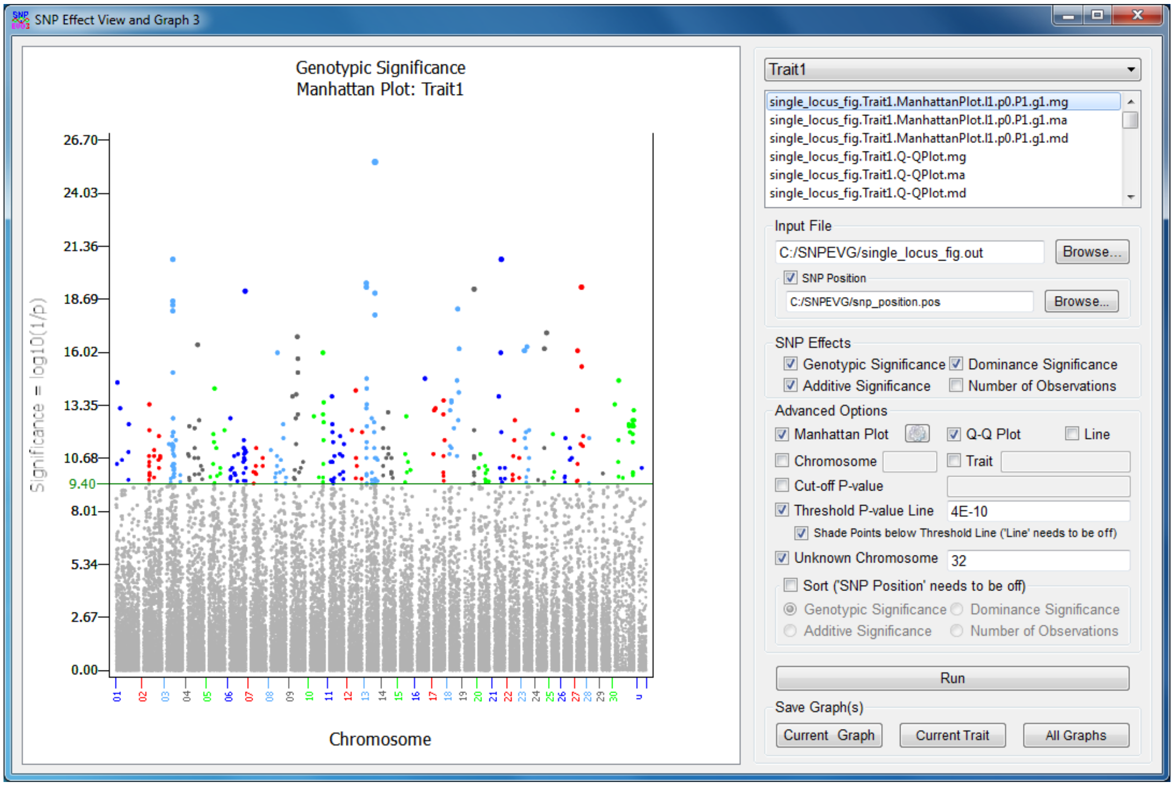


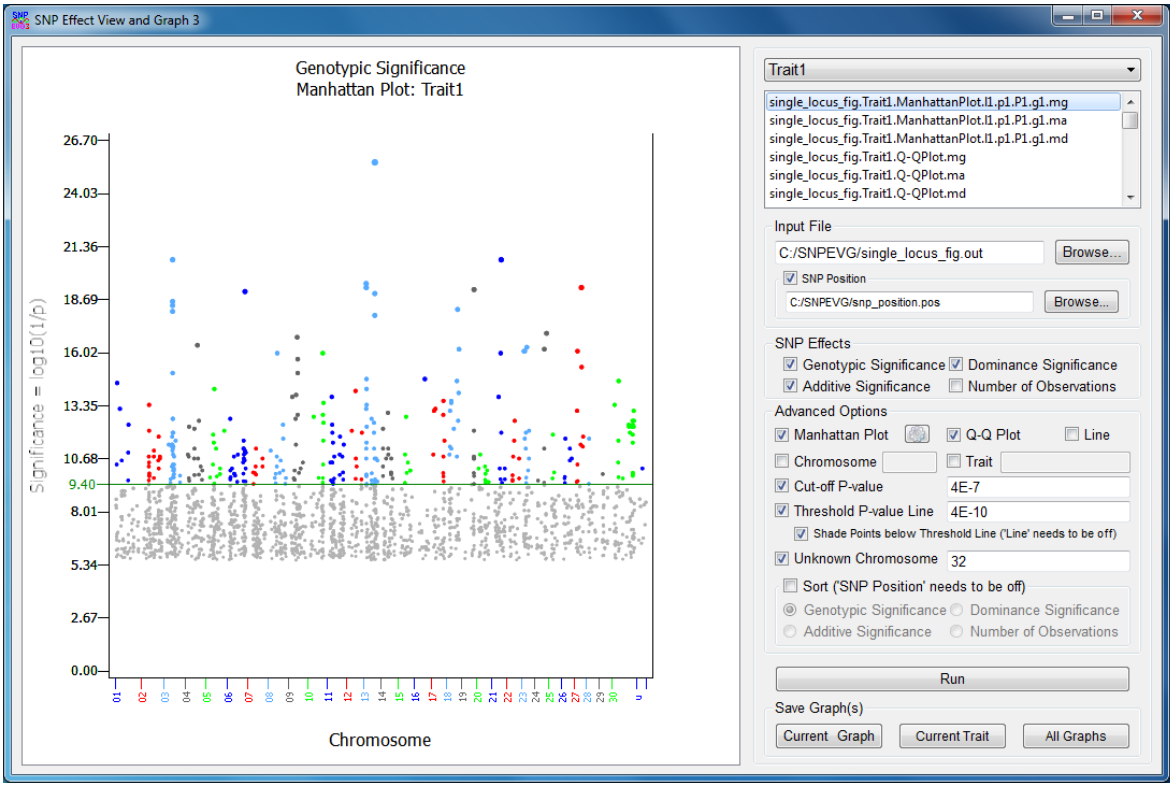


**Figure 5. Manhattan plot with shading of data points below threshold P-value line (upper) and Manhattan plot with cut-off P-value and shading of data points below threshold P-value line (lower).**

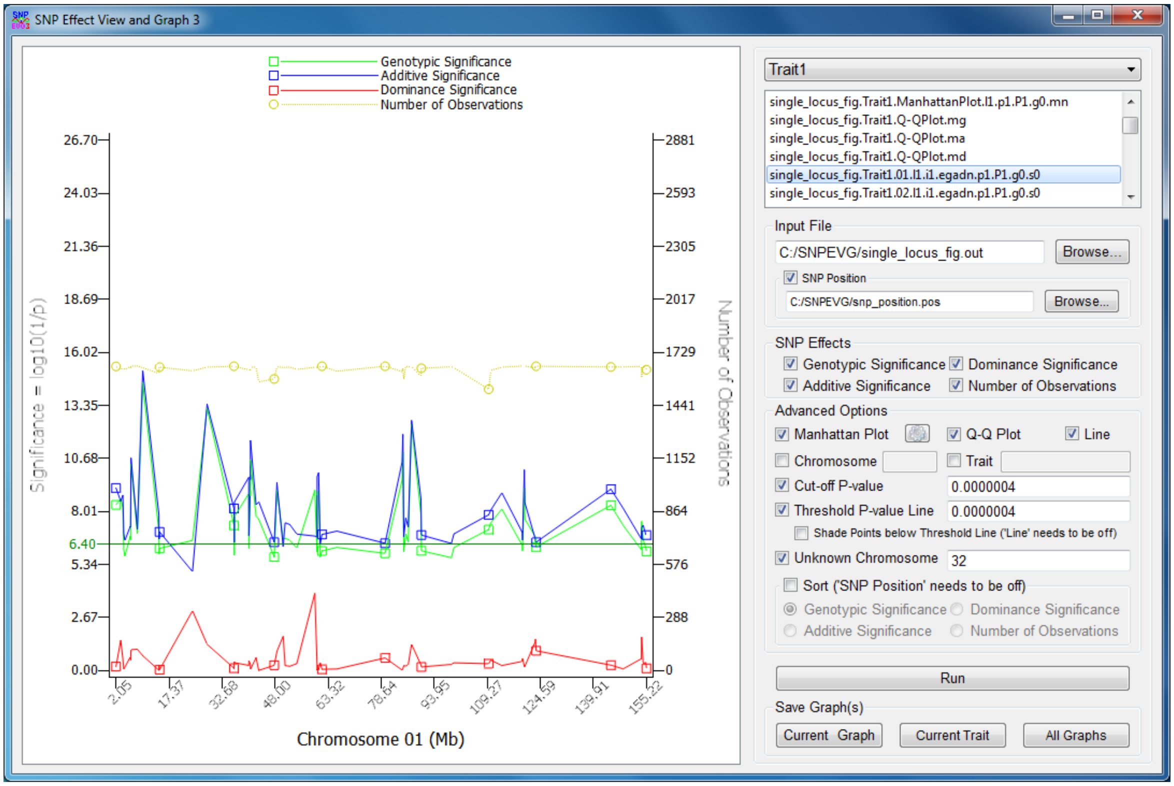


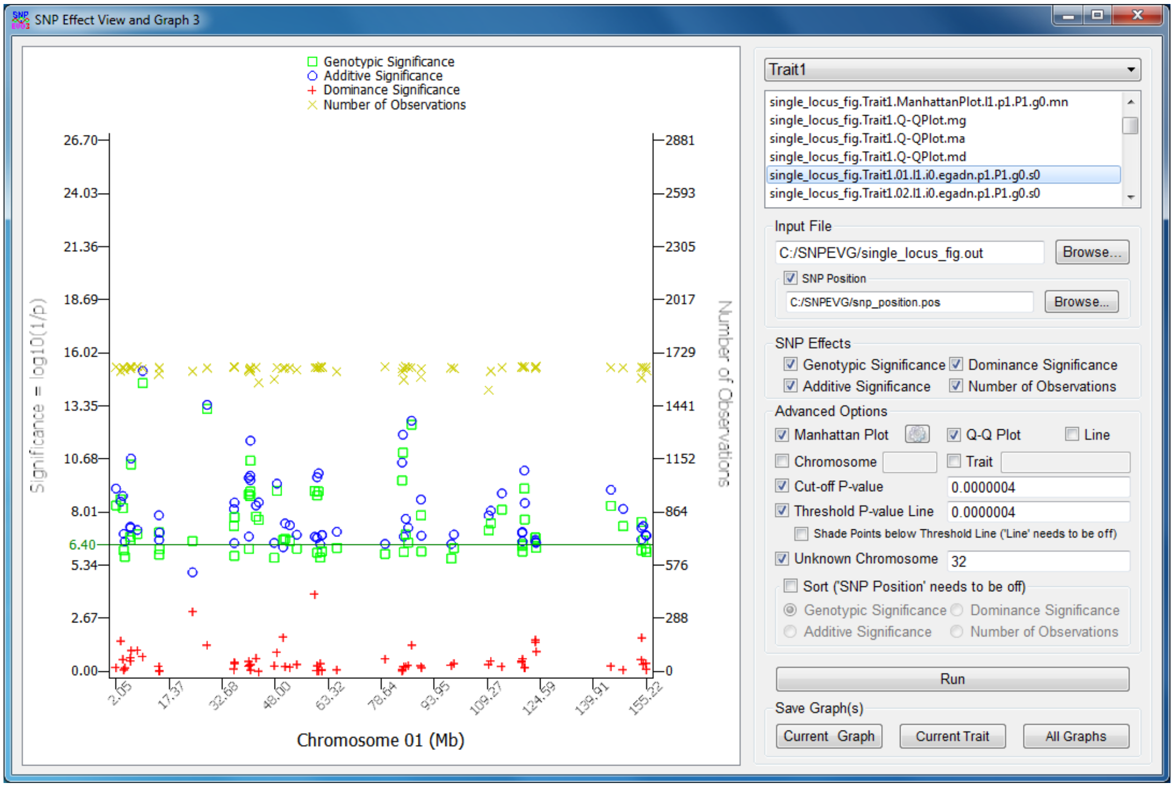


**Figure 6. Example of chromosome graph with cut-off P-value and threshold P-value, using lines (upper) or symbols (lower).**

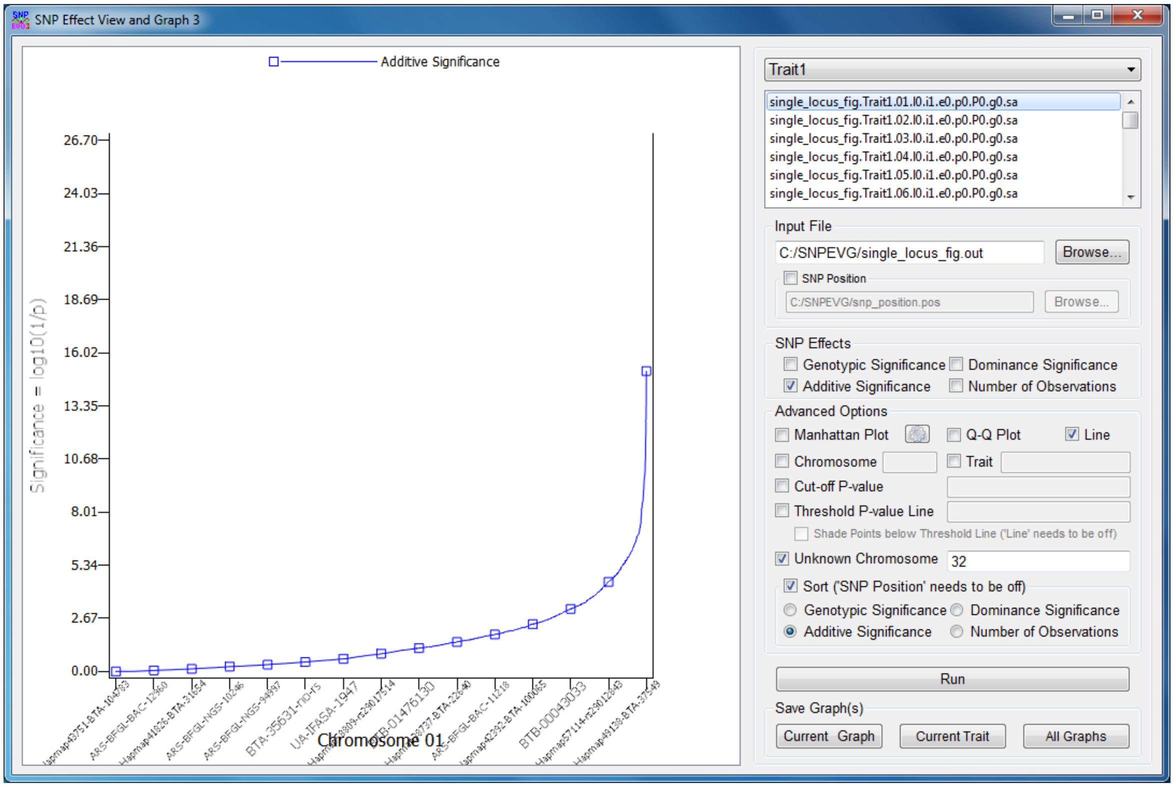


**Figure 7. SNP effects are sorted according to the significance of one effect, given that the position option is turned off.**

### 4.2 Chromosome Graphs by SNPEVG3

By default, each chromosome graph by SNPEVG3 displays three genetic effects (genotypic, additive and dominance effects) and the sample size for each SNP. All options for chromosome figures of SNPEVG1 are available for SNPEVG3. Examples are shown in **Figures 6**.

### 4.3 Sort SNP Effects (with Position Option off)

SNP effects can be sorted according to the significance of one effect, given that the position option is turned off (**Figure 7**).

### 4.4 Save Graphs to 'png' files

The program saves a graph to an image in the same folder of the input file by the graph's actual dimension. A selected graph can be saved to a 'png' file by clicking 'Current Graph', all the graphs of the currently selected trait can be saved to 'png' files by clicking 'Current Trait', and all the graphs of all traits can be saved to 'png' files by clicking 'All Graphs'.

# AUTHOR CONTRIBUTIONS

**Shengwen Wang** is the author of the SNPEVG3 program
**Daniel Dvorkin** is the author of the EPISNPPLOT program that is partially used in SNPEVG3
**Yang Da** is the project leader and the lead writer of this user manual

# RELEASE HISTORY

SNPEVG version 3.2: Released by S. Wang, D. Dvorkin and Y. Da, November 2012.

SNPEVG version 3.1: Released by S. Wang, D. Dvorkin and Y. Da, June 2012.

SNPEVG version 2.1: Released by S. Wang, D. Dvorkin and Y. Da, June 2011.

SNPEVG version 1.1: Released by S. Wang, D. Dvorkin and Y. Da, February 2011.

# REFERENCES


1. Ma L, Runesha HB, Dvorkin D, Garbe JR, Da Y: Parallel and serial computing tools for testing single-locus and epistatic SNP effects of quantitative traits in genome-wide association studies. *BMC Bioinformatics 2008*, 9:315–323.

2. Zhao JH: gap: Genetic analysis package. *J Stat Softw 2007*, 23(i08).
